# How Many Fish Need to Be Measured to Effectively Evaluate Trawl Selectivity?

**DOI:** 10.1371/journal.pone.0161512

**Published:** 2016-08-25

**Authors:** Bent Herrmann, Manu Sistiaga, Juan Santos, Antonello Sala

**Affiliations:** 1 SINTEF Fisheries and Aquaculture, Fishing Gear Technology, Willemoesvej 2, 9850, Hirtshals, Denmark; 2 SINTEF Fisheries and Aquaculture (SFA), Brattørkaia 17C, N-7010, Trondheim, Norway; 3 Thünen Institute for Baltic Sea Fisheries, Alter Hafen Süd 2, Rostock, 18069, Germany; 4 National Research Council (CNR) – Institute of Marine Sciences (ISMAR), Ancona, Largo Fiera della Pesca – 60125, Ancona, Italy; 5 Norwegian College of Fishery and Aquatic Science, University of Tromsø, 9037 Breivika, Tromsø, Norway; Sveriges lantbruksuniversitet, SWEDEN

## Abstract

The aim of this study was to provide practitioners working with trawl selectivity with general and easily understandable guidelines regarding the fish sampling effort necessary during sea trials. In particular, we focused on how many fish would need to be caught and length measured in a trawl haul in order to assess the selectivity parameters of the trawl at a designated uncertainty level. We also investigated the dependency of this uncertainty level on the experimental method used to collect data and on the potential effects of factors such as the size structure in the catch relative to the size selection of the gear. We based this study on simulated data created from two different fisheries: the Barents Sea cod (*Gadus morhua*) trawl fishery and the Mediterranean Sea multispecies trawl fishery represented by red mullet (*Mullus barbatus*). We used these two completely different fisheries to obtain results that can be used as general guidelines for other fisheries. We found that the uncertainty in the selection parameters decreased with increasing number of fish measured and that this relationship could be described by a power model. The sampling effort needed to achieve a specific uncertainty level for the selection parameters was always lower for the covered codend method compared to the paired-gear method. In many cases, the number of fish that would need to be measured to maintain a specific uncertainty level was around 10 times higher for the paired-gear method than for the covered codend method. The trends observed for the effect of sampling effort in the two fishery cases investigated were similar; therefore the guidelines presented herein should be applicable to other fisheries.

## Introduction

The development of the selective properties of fishing gears towards desired species or multispecies specific size selectivity is a widely used approach to attempt to achieve more sustainable fisheries [1]. Therefore determining the size selective properties of fishing gears is important for fisheries management. Mostly, the estimation and optimization of the size selective properties of fishing gears has been carried out based on analyzing data collected from experimental fishing. This is the case also for trawls, which represent one of the most important fishing methods used worldwide. The most basic measure of the size selective performance of a trawl is quantification of the size selectivity of the different species captured by the gear in individual hauls. The most common procedures applied to assess size selectivity in trawls and other towed fishing gears are outlined in cooperative research report no. 215 titled "Manual of methods of measuring the selectivity of towed fishing gears" [2].

The assessment of size selectivity in trawls has traditionally focused on the codend, and the majority of scientific studies conducted on codend size selectivity apply a size selection model in which the probability that a fish will be retained by the gear increases with increasing fish size. The model most often applied in these studies is the logit model [2]. Numerous examples of the application of the logit size selection model to describe size selectivity in trawl codends can be found in the literature (e.g., [3–14]). The quantity and diversity of studies that have used this model demonstrate its relevance and suitability for size selectivity research.

As described in Wileman et al. [2], the logit size selection model for a single haul can be fully described by two parameters, L50 (the length of fish with 50% probability of being retained by the gear) and SR (difference in length of fish with respectively 75% and 25% probability of being retained by the gear). Thus, many size selectivity studies for trawls include the assessment of L50 and SR for individual hauls. In scientific studies, an important aspect of assessing the value of model parameters such as L50 and SR is the assessment of the uncertainty in the values, often quantified by the 95% confidence intervals (CI's). Without the CI's, the parameter estimates themselves have little value as the CI's define the limits for the advice fishing gear scientists and fisheries managers can provide based on the analyzed size selection data. The assessment of the uncertainty in L50 and SR at haul level is also of essential importance even when this assessment is only a middle step to finally estimate the mean size selection for a group of hauls. The model of Fryer [15] for example, which has been widely used in selectivity studies, requires an initial analysis step in which the selection parameter values and uncertainties for each haul need to be estimated in terms of the parameters' covariance matrix [2, 15]. Hence, the estimation of the uncertainty of L50 and SR in individual hauls based on a logit selection model can be considered as an important aspect for the majority of trawl size selectivity studies. This leads to a number of questions related to how many fish would need to be caught and measured in a typical trawl haul to obtain a given level of uncertainty of L50 and SR (e.g., within ±1%, 2%, 5%, or 10% of the true value). To address an issue such as the uncertainty in the selectivity parameters L50 and SR, we first need to consider how size selection data are typically collected during an experimental trawl haul. The covered codend and paired-gear methods are the two main experimental methodologies applied to collect trawl selectivity data [2]. In the covered codend method, fish escaping from the gear are retained in one or more small mesh covers, which enables direct estimation of the fish retained in the codend and the fish that escaped through the gear during the haul. The twin trawl, trouser trawl, parallel haul and alternate haul methods are all classified by Wileman et al. [2] as paired-gear methods. In these methods, the fish retained by the gear is directly estimated from the fish retained by the test codend, whereas the population fished on is indirectly estimated from a small mesh control gear that is towed simultaneously on one side of the twin/trouser trawl, from a different vessel (parallel), or by the same vessel (the latter would alternate hauls between test and control gears). The indiscriminate use of these two different experimental sampling methods lead to questions related to their efficiencies of the methods compared to each other in terms of the number of fish that need to be measured to keep the uncertainty of the estimated selection parameters within specific limits.

Often the quantity of fish caught in each of the gear compartments (the codend and covers or control) in a trawl haul is so big that it is practically impossible to length measure all fish or it is simply not considered necessary. In such cases, a subsample of fish is length measured from each of the compartments, and the rest of the catch is counted or weighed to calculate the subsampling ratio [2]. The aspect of subsampling leads to additional questions related to whether the quantity of fish being length measured is high enough to be able to estimate the selection parameter values within specific acceptable limits of uncertainty. This question is relevant in the field because the answer determines when to stop length measuring fish from the collected catch.

General guidelines about the number of fish that would need to be caught and length measured in a trawl haul to assess the selection parameters within an acceptable range of uncertainty, how this depends on the experimental method applied for data collection, and the potential effects of factors such as the size structure in the catch relative to the size selection of the gear would be a valuable tool for gear technologists. Such a tool would help scientists plan selectivity trials to get most out of the often limited and expensive cruise time, and judge the consequences of using one or the other experimental method. Thus, the objective of the current study was to develop and communicate general and easily understandable guidelines for practitioners working with trawl selectivity trials.

## Materials and Methods

### Assessment of 95% CIs for selection parameters obtained for individual hauls

The estimation of the selection parameters L50 and SR for a trawl haul usually are based on a maximum likelihood fit of a size selection model to the experimental data. The experimental selection data for an individual haul and a certain species consist of count data for the number fish retained in each of the gear compartments (codend and covers or control) sorted into so-called length classes [2]. For the covered codend sampling method only the parameters L50 and SR need to be estimated by fitting the size selection model to the size selection data. For the paired-gear method an additional parameter known as the split parameter (SP), which quantifies the fraction of fish (from the total amount entering the gear) that enters the test gear, also needs to be estimated when fitting the model to the data [2]. SP is a nuisance parameter that provides no information about the selection properties of the gear tested, but it needs to be estimated together with L50 and SR when using the paired-gear sampling method [11]. This estimation procedure for the paired-gear data is also known as the SELECT (Share Each LEngth Catch Total) method and it was first formally described in [16]. The SELECT analysis method can be seen as a generalization of the covered codend analysis as in both cases is the observed catch sharing between gear compartments modelled based on a binomial assumption. In case of the covered codend between the test codend and the surrounding cover and in case of the paired-gear method between the test codend and the small mesh control codend.

The CIs for the selection parameters are estimated based their covariance matrix which is obtained based on the maximum likelihood estimation following the procedure outlined in Wileman et al. [2]. The diagonal elements in the covariance matrix represent the variance estimates for the selection parameters. When there is an indication of overdispersion in the data, the estimated variances are adjusted for this before the next step in the estimation of the CIs (see Wileman et al. [2] for details). Based on the parameter variance, the 95%CIs are then calculated as the estimated parameter value ± the square root of the parameter variance multiplied by the factor *t(DOF)*, which is a t-quantile obtained based on a t-distribution [17]. DOF represents the degrees of freedom and is calculated as the number of length classes in the experimental selection data minus the number of model parameters estimated. For a double sided 95% CI, *t(DOF)* is a decreasing function of DOF with 1.96 as the asymptotic value. The values for each specific DOF value can be found in several textbooks of basic statistics (e.g., Rees [17]).

Because the procedure outlined above is often applied to estimate the CIs for the selection parameters in trawl selectivity studies, we quantified the expected uncertainty of the selection parameter values obtained from individual trawl hauls using this approach. Specifically, we quantified the uncertainty in L50 and SR in % by *delL50* and *delSR*, which we calculate by:

$$\begin{array}{c} delL50=\pm \frac{t(DOF)\times \sqrt{varL50}\times 100}{L50}\\ delSR=\pm \frac{t(DOF)\times \sqrt{varSR}\times 100}{SR} \end{array}$$ (1)

The analysis of size selectivity data for single hauls was carried out as described above using the software tool SELNET [18].

### Simulation of size selectivity data for the different fisheries and fishing scenarios

To investigate the potential effects of the size structure of the population fished (and therefore also of the collected size selection data) on the uncertainty of the estimated selection parameters, we used simulated size selection data. The use of simulated data enabled us to make systematic and well-controlled changes to the data to compare the performance of the covered codend and paired-gear sampling methods. To simulate realistic and relevant size selection data, we used L50 and SR values estimated from two real hauls from two important but very different European trawl fisheries as initial points for the simulations. By using two completely different fisheries we hoped that the results obtained could serve as guidelines for other fisheries as well.

We investigated if the uncertainty of the estimated L50 and SR values followed the same pattern for two different fisheries. We chose the Barents Sea bottom trawl cod (*Gadus morhua*) fishery [19] and the Mediterranean multispecies bottom trawl fishery [8] with red mullet (*Mullus barbatus*) as the reference species. From each of these two fisheries we handpicked a single haul that we believed represented the fishery well. In both cases a large number of fish from the haul had been measured, which enabled the estimation of the size selection parameters with low uncertainty. For both fisheries the data were collected as covered codend data, with 1075 and 1108 cod and 720 and 546 red mullet in the codend and the cover, respectively. There was no subsampling of the data. For cod the selectivity data were collected in 1.0 cm length classes, which is a length class width typically used in selectivity trials in North European fisheries. For red mullet the data were collected in 0.5 cm length classes, which is typical for many selectivity trials in the Mediterranean area. For further information about these two hauls see references [19] and [8].

**Fig 1:**
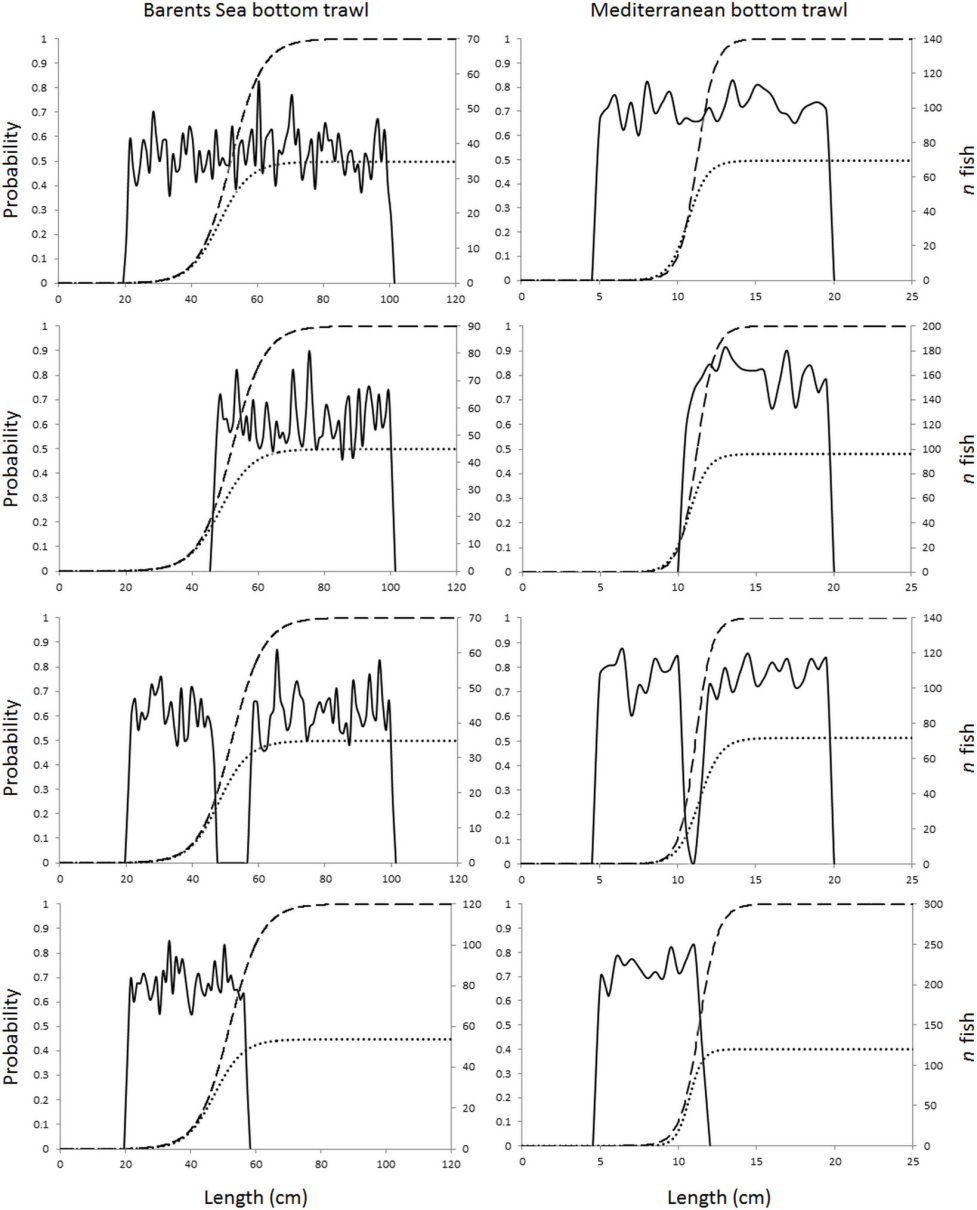
Four different fish size distribution scenarios considered in the simulation (from top): uniform, no small, no medium, and no big fish. The stippled curve represents the simulated selection curve, and the dotted curve represents the corresponding paired-gear curve for SP = 0.5. The plots in the left column belong to the Barents Sea bottom trawl cod fishery, and the plots in the right column belong to the Mediterranean bottom trawl fishery represented by red mullet.

The data for the two handpicked hauls were analyzed by fitting a logit selection model to the experimental data. In both cases the logit model described the data well, as the p-value, which quantifies the probability to by coincidence obtain at least as big discrepancy between data and model, was 0.52 for the Barents Sea haul and 0.76 for the Mediterranean Sea haul. Based on this analysis of the data, the selection parameters used for the Barents Sea cod were L50 = 52.00 cm and SR = 10.50 cm, and those for the Mediterranean Sea red mullet were L50 = 11.25 cm and SR = 1.25 cm. Using these L50 and SR values we simulated size selection data for a covered codend haul and a paired-gear haul for each of the two fisheries and for four different population structure scenarios (i.e., structure in the fished population): (i) uniform population; (ii) no small fish (no fish < L25); (iii) no medium fish (no fish between L25 and L75); and (iv) no big fish (no fish > L75). To simulate the paired-gear selection data we used an SP value of 0.5, which assumes equal entry of fish to the test and control codends. The selectivity data were simulated assuming a logit selection model using the parametric simulation facilities built into the software tool SELNET [20]. We used a fished population of 3000 fish in all cases, which later enabled us to investigate the effect of the number of fish measured on the uncertainty of L50 and SR by subsampling of the simulated haul data. Fig 1 illustrates the population scenarios considered for the two cases.

### Simulation of subsampled size selection data

**Fig 2:**
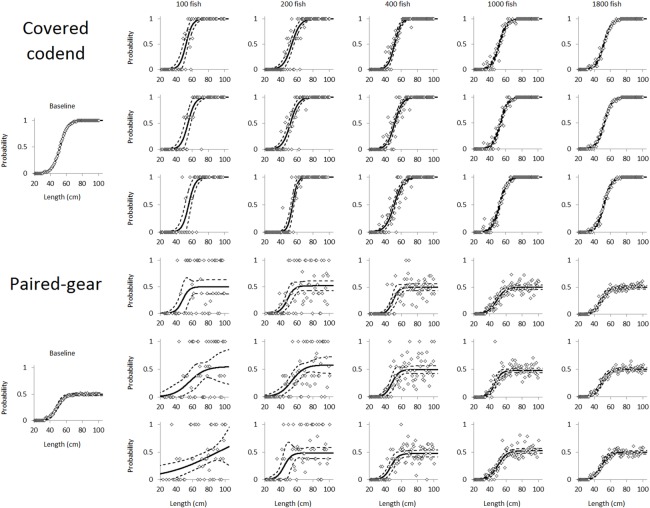
Illustration of the random subsampling of size selectivity data for the covered codend method (above) and the paired-gear method (below). For each of the sampling levels, 100, 200, 400, 1000, and 1800 fish measured and three different simulated hauls out of the 1000 simulated hauls are shown for each situation. This illustration is based on the uniform size distribution for the Barents Sea bottom trawl cod fishery, but the procedure was identical for all other cases. The white marks in the plots represent the experimental points for size selection directly (covered codend method) and indirectly through the paired curve (paired-gear method). The solid curves represent the size selection curve and the paired curve fitted to the data. The stippled curves represent the estimated 95% confidence limits for the curves.

Based on the size selectivity data simulated for a single haul for each different scenario, we investigated the effect of sampling size (in terms of number of fish length measured) on the uncertainty of L50 and SR for each case separately. Thus, we simulated different subsampling levels for each fishery and each of the four investigated scenarios. We fixed the subsampling levels to measurements of equal numbers of fish from each of the two existing compartments: the codend and cover in the case of the covered codend method and the codend and control in the case of the paired-gear method. We chose this sampling strategy because Millar [21] reported that it had the best overall performance among the different methods he explored. For each case we simulated sample size starting at 50 fish in each compartment (a total of 100 fish measured) and increased it in steps of 50 fish to the maximum possible number that included an equal number of fish from each compartment. Random sampling was conducted compartment by compartment separately and without replacement to mimic a real case of subsampling. At each sampling level we simulated 1000 different subsampled hauls using a random sampling procedure. Analyzing these simulated 1000 haul sets enabled us to estimate the expected uncertainty for L50 and SR at each sampling level and for each fishery case and fishery scenario separately. The simulation of this subsampling procedure was conducted applying simulation facilities in the software tool SELNET. For the cod fishery the size selection data were formatted using 1.0 cm length classes, whereas for the red mullet data the length class width used was 0.5 cm. These length class widths represent the typical measuring procedures used in each fishery. Fig 2 illustrates the sampling and analysis procedure used in this study; it shows three randomly chosen hauls for both the covered codend method and the paired-gear method for sampling levels of 100, 200, 400, 1000, and 1800 length measured fish in a population structure scenario with a uniform size distribution of cod.

### Assessment of uncertainty of L50 and SR for each sampling set

Each subsampled haul in each sampling set of 1000 hauls (section 2.3) was analyzed by fitting a logit selection model to the data. Thus, for each sampling set we estimated 1000 values of L50 and SR with their corresponding uncertainties *delL50* and *delSR* (following Eq (1)). The analysis was conducted using the SELNET software. Based on the 1000 results obtained for each sampling set we estimated the mean value of the obtained L50, SR, *delL50*, and *delSR* and used these mean values as the expected values for each subsampling level. This analysis was carried out for each set of 1000 hauls at each subsampling level, for the covered codend and paired-gear data, and for each population structure scenario for both fishery cases (cod and red mullet). In addition to the mean values, the 2.5 and 97.5 percentile values for *delL50* and *delSR* were also estimated for each sampling set so that the 95% CIs for the uncertainty in selection parameter uncertainties could be calculated at each sampling level. In most cases it was possible to obtain parameter values for each of the 1000 simulated hauls analyzed for each subsampling level, but in a few cases some results had to be eliminated from the analysis because the covariance matrix could not be estimated.

The estimated mean values of *delL50* and *delSR* for each population size structure scenario were plotted against the number of fish length measured to illustrate how the expected uncertainty in the selection parameters depends on the number of fish measured. To obtain predictions outside the specific subsampling levels simulated we fitted the following power model to the data using a least squares estimation method:

$$mean(\;delX(n))=a\times n^{b}$$ (2)

where X stands for L50 or SR, *n* is the sample size (two times the number of fish measured in each compartment), and *a* and *b* are the parameters to be estimated. The R^2^ value (the fraction of variance in the data explained the model) [22] was used as a measure to quantify the ability of the model (2) to describe the dependency of the expected uncertainty in the selection parameter as a function of the number of fish measured.

The relative efficiency *e(n)* of the covered codend (*CC*) compared to the paired-gear (*PG*) sampling method regarding the uncertainty in the selection parameter values was assessed individually for each population structure scenario by:

$$e(n)=\frac{mean(PG\_delX(n))}{mean(CC\_delX(n))}$$ (3)

An *e(n)* value of 1.0 would mean that the two sampling methods are equally efficient, whereas a value > 1.0 would mean that the paired-gear method is less efficient.

The current study focused on investigating the uncertainty in L50 and SR, but the mean L50 and mean SR values obtained for each sampling set also enabled us to check for potential bias in the estimated selection parameters when sample sizes were decreased (measuring fewer fish). Because the study was based on simulation, we did know the true L50 and SR values. Therefore, we could calculate the bias at each sampling level simply by subtracting the estimated mean value from the true parameter value. By dividing this bias by the true value and multiplying it by 100 we obtained the bias percentage. This enabled us to evaluate whether the bias in percentage was within the estimated uncertainty in percentage for the selection parameters, which would mean that the bias was not significant.

### Assessment of the number of fish that would need to be length measured to obtain a specific level of expected uncertainty in L50 and SR

Based on *a* and *b* (Eq (2)) for each of the cases investigated and for each of the two sampling methods individually we could obtain a direct prediction of the expected number of fish *n* that must be caught and length measured to obtain an uncertainty in mean *delL50* and mean *delSR* that would not exceed a specific limit. We obtained this prediction by solving Eq (2) with respect to *n*:

$$n(mean(delX))={\left(\frac{mean(delX)}{a}\right)}^{\frac{1}{b}}$$ (4)

Using Eq (4) for each case separately, we predicted the number of fish that would need to be length measured as a function of the intended uncertainty in L50 and SR. To compare the two sampling methods, it was also relevant to estimate the ratio between the number of fish that would need to be measured for each method to obtain a specific uncertainty in the selection parameters. Based on Eq (4) this prediction could be obtained as follows:

$${ratio}_{n}(mean(delX))=\frac{PG\_n(mean(delX))}{CC\_n(mean(delX))}$$ (5)

For example, if ratio_n_ is 3.0 for a given population structure scenario and a specific intended uncertainty, the number of fish that would need to be measured when using the paired-gear sampling method is three times greater than that when using the covered codend sampling method.

### Using single haul estimate uncertainties as a guideline for the uncertainty in mean selection based on multiple hauls

Estimation of the uncertainty in individual haul selection parameters is often a middle step for the final objective of estimating the mean size selection based on a group of hauls conducted with the same gear. If the size selection of a gear varies very little between individual hauls then a valid estimate of the uncertainty for the mean selection parameters for that group of hauls based on the total number of fish measured in them can be approximated by results obtained following the procedures described in the previous sections by using the total number of fish measured for the group of hauls as the number of fish in the analysis. However, often size selection can be expected to vary between hauls, and as demonstrated by Fryer [15], a simple analysis based on data pooled over hauls without considering the effect of between-haul variation in size selection can lead to underestimation of the uncertainty in the mean size selection. In such cases, the uncertainty estimated in the present single haul investigation can be applied as a lower limit guideline for the level of uncertainty that can be obtained with the total number of fish measured in the group of hauls. Thus, we propose that the results presented in this single haul approach can be applied as a guideline for the minimum expected uncertainty in mean selection parameter values that can be obtained for a group of hauls with a specific total number of fish being length measured.

## Results

### Uncertainty in selection parameters for different numbers of fish measured

**Fig 3:**
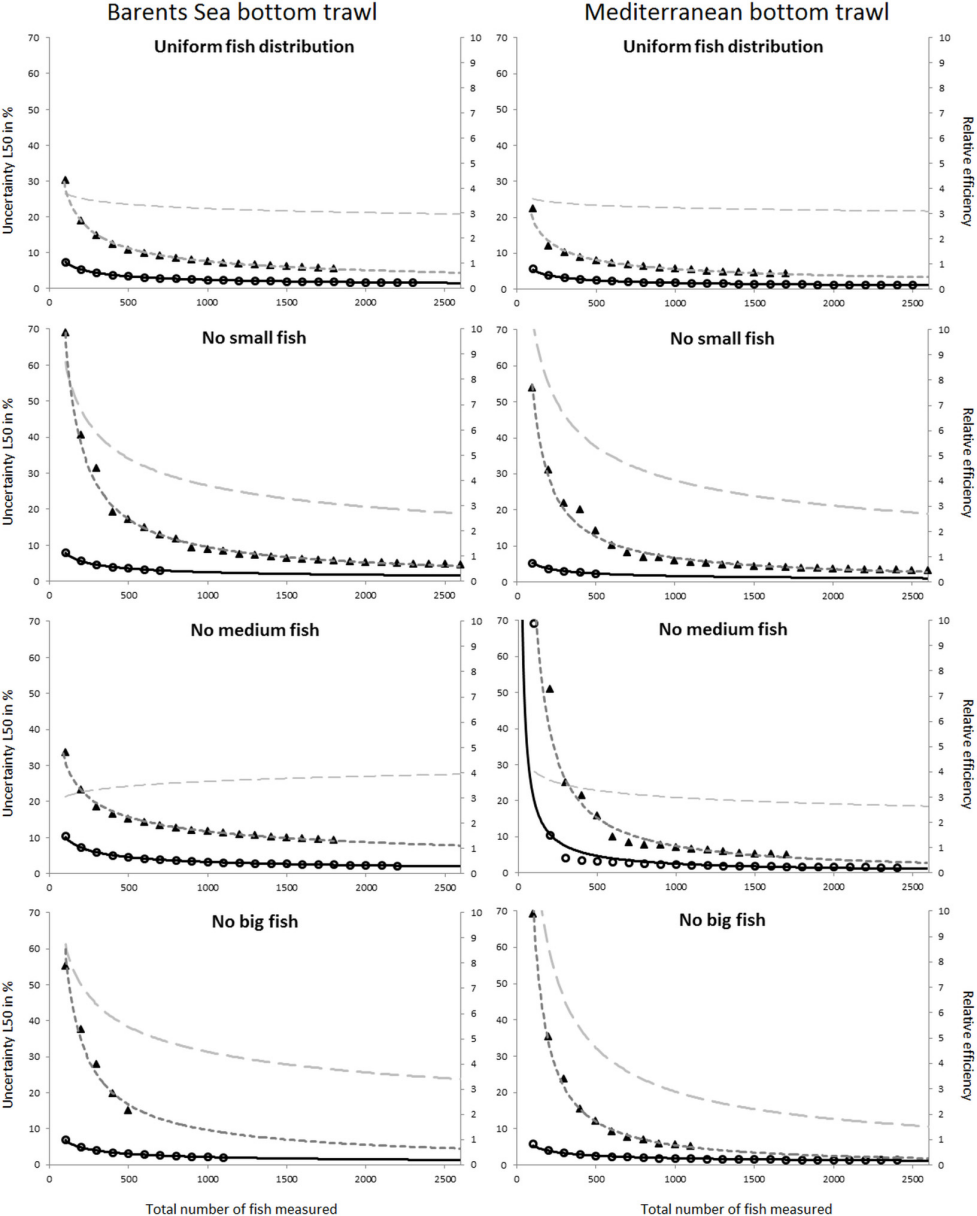
Predicted uncertainty for L50 in % (left axis) versus number of fish length measured for the covered codend method (circles) and the paired-gear method (triangles). The solid black curve is the power model (2) fitted to the points for the covered codend data, and the stippled black curve represents the same for the paired-gear method. The grey stippled curve is the relative efficiency (right axis) between the paired-gear and the covered codend methods calculated according to Eq (3). Results are shown for the four different fish size distributions considered (from top): uniform, no small, no medium, and no big fish. The left column shows predictions for the Barents Sea bottom trawl cod fishery, and the right column shows predictions for the Mediterranean bottom trawl fishery represented by red mullet.

**Fig 4:**
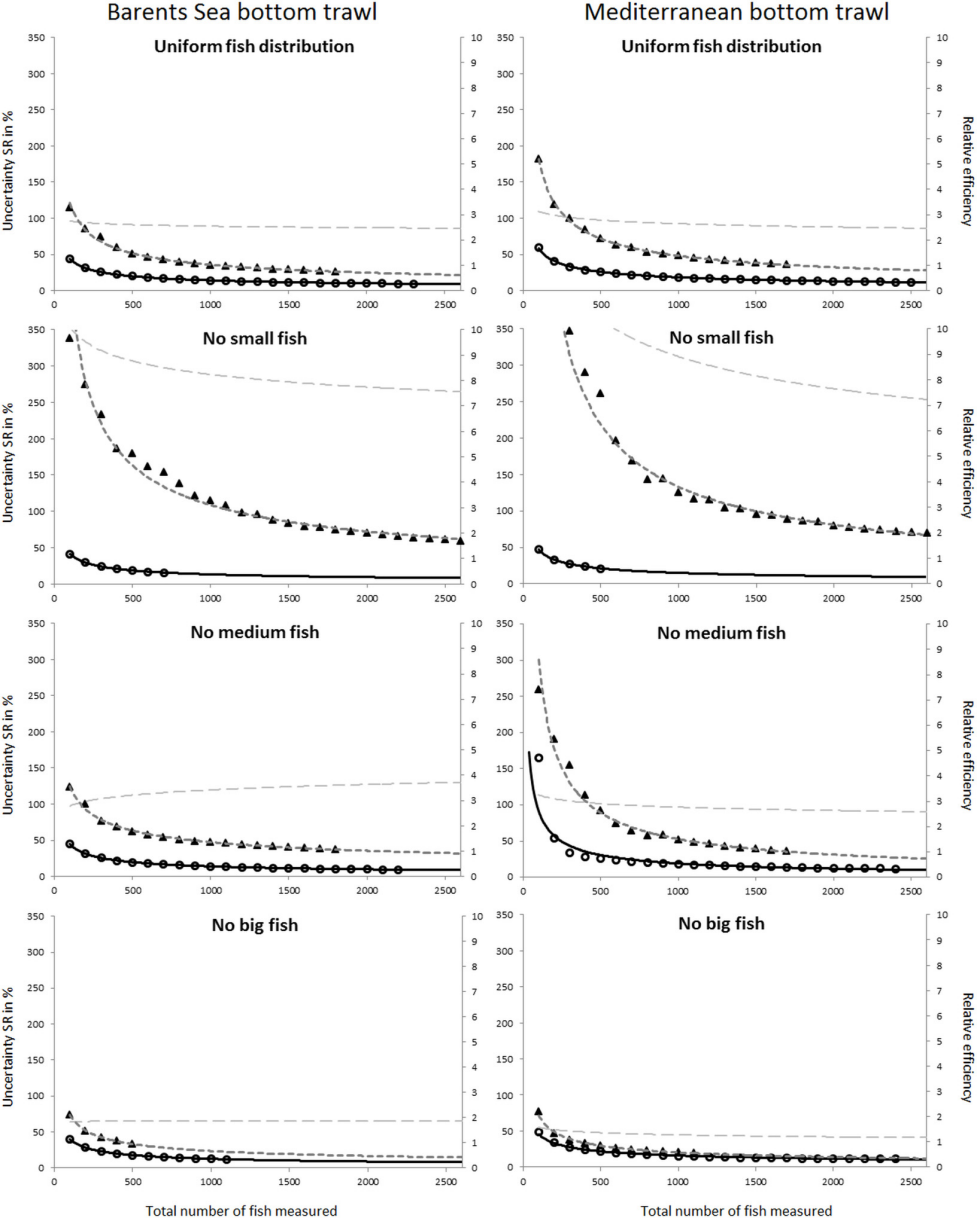
Predicted uncertainty for SR in % (left axis) versus number of fish length measured for the covered codend method (circles) and the paired-gear method (triangles). The solid black curve is the power model (2) fitted to the points for the covered codend data, and the stippled black curve represents the same for the paired-gear method. The grey stippled curve is the relative efficiency (right axis) between the paired-gear and the covered codend methods calculated according to Eq (3). Results are shown for the four different fish size distributions considered (from top): uniform, no small, no medium, and no big fish. The left column shows predictions for the Barents Sea bottom trawl cod fishery, and the right column shows predictions for the Mediterranean bottom trawl fishery represented by red mullet.

The uncertainty in the selection parameters L50 and SR was assessed for both the Barents Sea and the Mediterranean fisheries, for all four population structure scenarios considered, and for both the covered codend and paired-gear sampling methods. Fig 3 shows the uncertainty in L50 versus number of fish length measured for all studied cases. A similar plot for the uncertainty in SR is shown in Fig 4. The results used to produce the plots in Figs 3 and 4 are included in the eight tables shown in Tables F–M in S1 Appendix.

In general the expected uncertainty for both L50 and SR was greater for the paired-gear sampling method (triangles in Figs 3 and 4) than for the covered codend sampling method (circles in Figs 3 and 4), and the trends were similar for the two fisheries. The power models fitted to the points in Figs 3 and 4 demonstrate that this type of model can describe well the decrease in uncertainty with increasing number of fish length measured for both L50 and SR. This premise is further supported by the high R^2^ values obtained for the different cases (Table 1). In fact, the R^2^ value was < 0.9656 in only one case.

**Table 1:** Least squares fit of the power model (2) describing the uncertainty in selection parameters versus number of fish length measured. a and b are the parameters in the model (2) and R^2^ is the ratio of variation explained by the model. Model fits are given for the different fish size distributions considered for both the Barents Sea bottom trawl fishery targeting cod (left) and the Mediterranean fishery bottom trawl fishery with red mullet as reference species (right). Model parameters are given for both the covered codend and the paired-gear methods.

			Barents Sea bottom trawl			Mediterranean bottom trawl		
			a	b	R^2^	a	b	R^2^
Uncertainty L50 (%)	Uniform fish distribution	Covered codend	72.672	-0.496	1.000	54.651	-0.501	1.000
		Paired-gear	388.44	-0.571	0.996	241.26	-0.546	0.988
	No small fish	Covered codend	79.395	-0.501	1.000	52.255	-0.499	1.000
		Paired-gear	415.47	-0.500	1.000	3677.5	-0.913	0.982
	No medium fish	Covered codend	103.32	-0.503	1.000	1292.7	-0.906	0.822
		Paired-gear	220.21	-0.425	0.992	9400.1	-1.035	0.974
	No big fish	Covered codend	69.146	-0.502	1.000	44.481	-0.458	0.990
		Paired-gear	2297.6	-0.792	0.966	13552	-1.132	0.995
Uncertainty SR (%)	Uniform fish distribution	Covered codend	431.41	-0.496	1.000	587.17	-0.503	0.999
		Paired-gear	1386.9	-0.530	0.995	2587.2	-0.577	0.999
	No small fish	Covered codend	426.26	-0.508	1.000	458.32	-0.496	1.000
		Paired-gear	6374	-0.590	0.980	18670	-0.716	0.986
	No medium fish	Covered codend	426.26	-0.508	1.000	2172.7	-0.686	0.927
		Paired-gear	854.21	-0.419	0.995	9754.7	-0.756	0.987
	No big fish	Covered codend	405.61	-0.503	1.000	372.82	-0.457	0.991
		Paired-gear	726.35	-0.497	1.000	891.22	-0.548	0.993

Based on the models defined by the parameter values listed in Table 1, the relative efficiency of the covered codend compared to the paired-gear method was assessed using Eq (3). The curves estimated for the relative efficiency (stippled grey curves in Figs 3 and 4) show values ranging from 1.2 to ~10.0. This result illustrates that the paired-gear method is less efficient than the covered codend method in terms of the number of fish that need to be measured to obtain a certain level of uncertainty in the selection parameters.

**Fig 5:**
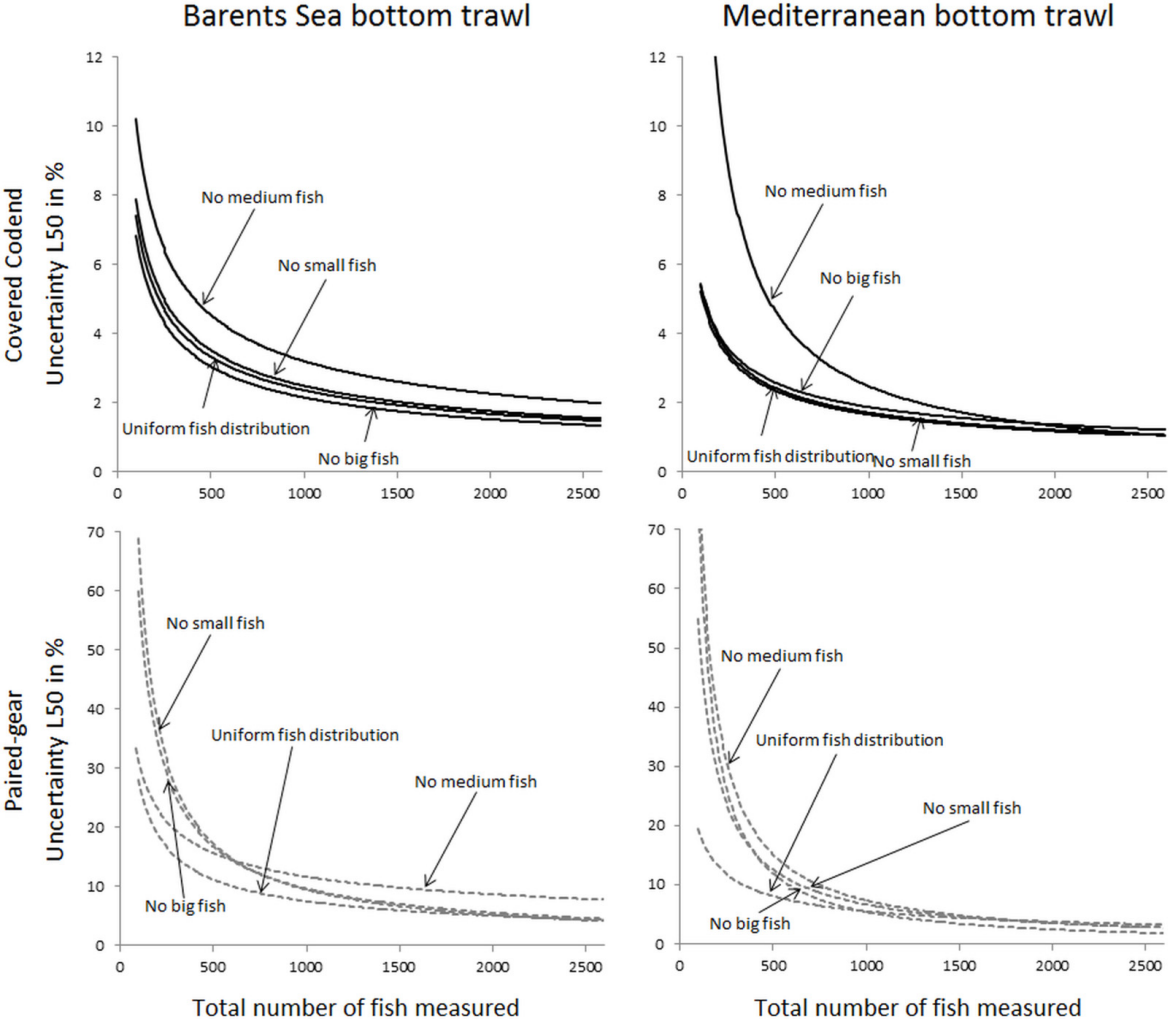
Comparison of the uncertainty in L50 (%) versus the number of fish measured for the four different fish size distribution scenarios. The first row includes predictions for the covered codend method, and the second includes predictions for the paired-gear method. The left column shows the results for the Barents Sea bottom trawl cod fishery, and the right column shows the results for the Mediterranean bottom trawl fishery represented by red mullet.

**Fig 6:**
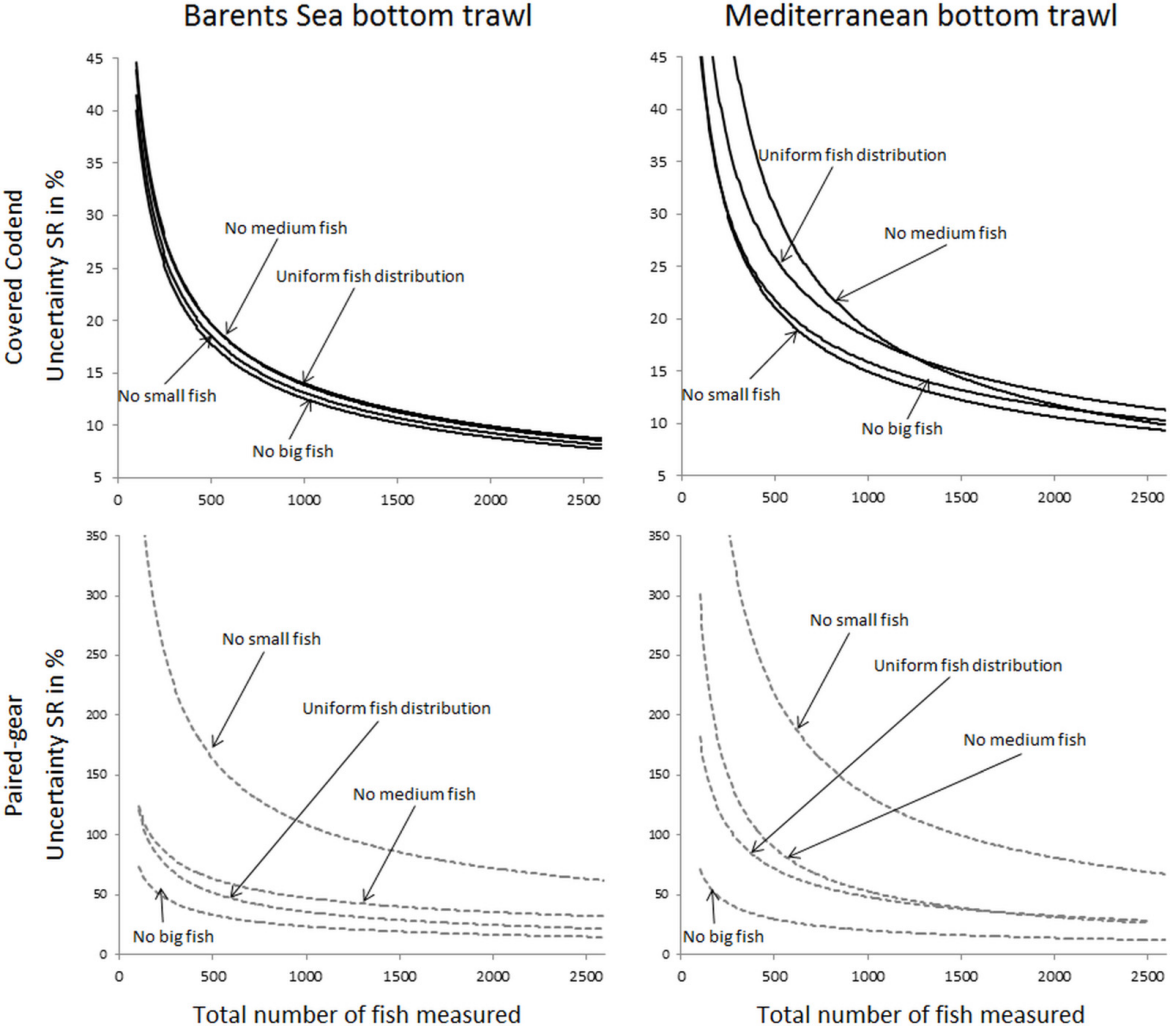
Comparison of the uncertainty in SR (%) versus the number of fish measured for the four different fish size distribution scenarios. The first row includes predictions for the covered codend method, and the second includes predictions for the paired-gear method. The left column shows the results for the Barents Sea bottom trawl cod fishery, and the right column shows the results for the Mediterranean bottom trawl fishery represented by red mullet.

Figs 5 and 6 compare the uncertainty obtained for L50 and SR for all studied cases. Although the two fisheries included in this study deal with different species, the results share similar tendencies. For example, the case that represents the covered codend method and fishing on a population structure lacking medium sized fish showed the highest uncertainty both for L50 and SR, with results being most profound for L50. For the paired-gear method the pattern was not as clear, but the lack of small fish led to much greater uncertainty in SR compared to all other fish distribution patterns studied for both fisheries.

The tables in S1 Appendix, which contain the results used to create the plots shown in Figs 3–6, enabled us to check for bias in the estimated L50 and SR values. Inspection of these results revealed that in general the estimated bias was within the expected uncertainty levels and therefore that the estimated bias was not significant. This was true in all cases except for a very few for the Mediterranean fishery using the paired-gear sampling method, where the bias in L50 just exceeded the expected uncertainty. For the cases with > 1500 fish measured with no medium sized fish in the population structure, the bias stayed inside the 95% distribution limits for the uncertainty. The only other case with significant bias was for SR for red mullet in the scenario with no big fish using the paired-gear sampling method. Here SR was very low in value (between 40 and 30%), and the bias was significant when > 400 fish were measured. In summary, significant bias occurred in only a very few cases with troublesome size distributions and only for the paired-gear sampling method.

### Number of fish that would need to be measured to obtain a specific uncertainty level in selection parameters

The main goal of this study was to provide guidelines about how many fish need to be length measured in a trawl haul to be able to estimate selection parameter values within a specific intended uncertainty level. Figs 3 and 4 show that this number definitely depends on the data collection method applied (paired-gear or covered codend). The type of fishery and the fish size distribution in the fishing area (Figs 5 and 6) also affect the number of fish that need to be measured but to a lesser extent than the sampling method.

**Fig 7:**
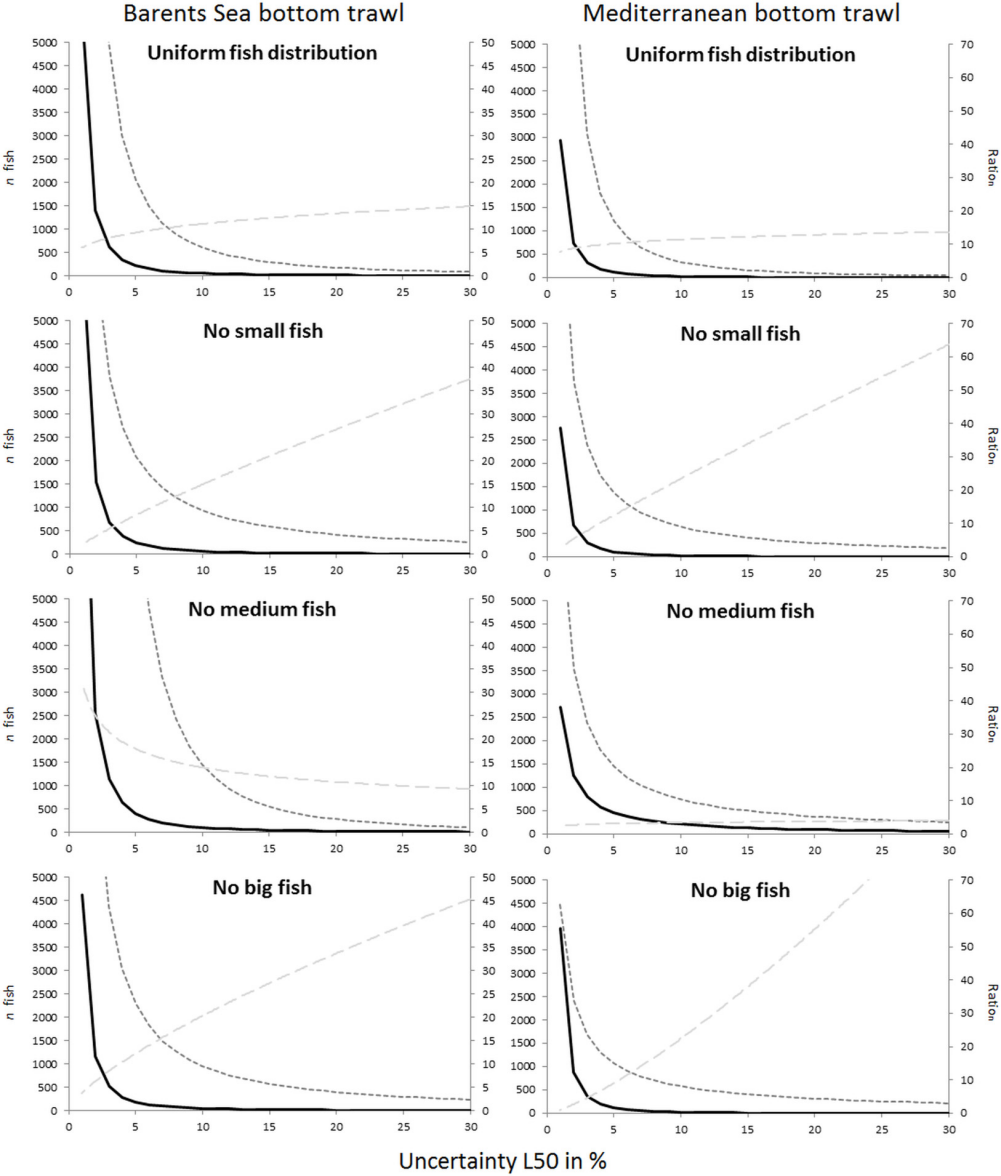
Predictions of number of fish that need to be measured to obtain an L50 value (left axis) within specific levels of uncertainty (%) for the covered codend sampling method (solid black curve) and the paired-gear sampling method (stippled black curve). The stippled grey curve quantifies the ratio in number of fish, ratio_n_ (right axis), that need to be measured with the paired-gear method compared to the covered codend method to obtain the same level of uncertainty in L50. Predictions are shown for the four different fish size distribution scenarios investigated (from top): uniform, no small, no medium, and no big fish. The left column shows the results for the Barents Sea bottom trawl fishery, and the right column shows the results for the Mediterranean bottom trawl fishery represented by red mullet.

For L50, Fig 7 and Tables 2 and 3 illustrate that the number of fish that needed to be length measured was always much higher for the paired-gear method than for the covered codend method. For an expected uncertainty of ± 5%, for example, the ratio_n_ for the cod fishery (Table 2) was 9.25, 12.29, 17.91, and 8.41, respectively, depending on the size distribution of fish entering the gear. This means that to obtain a ± 5% uncertainty level for L50 we would have to measure between 8.41 and 17.91 times as many fish with the paired-gear method as with the covered codend method. In actual numbers, these differences are represented, respectively, by 221 vs. 2045, 187 vs. 2299, 412 vs. 7378, and 249 vs. 2094. When the acceptable uncertainty level for L50 in the cod fishery was increased to ± 10%, the ratio_n_ ranged between 11.04 and 20.38, and the number of fish that needed to be measured ranged between 47 and 104 for the covered codend and between 607 and 1444 fish for the paired-gear method. Thus, the paired-gear sampling method requires length measurement of roughly 10 times as many fish as the covered codend method to obtain similar uncertainty in L50.

**Table 2:** Predicted number of fish needed to be measured to obtain an L50 value within a specific uncertainty (%) for the Barents Sea fishery targeting cod. Numbers are given for the covered codend and paired-gear sampling methods, and for each of the four fish size distribution scenarios investigated. Ratio_n_ quantifies the ratio of fish needed to be measured with the paired-gear sampling method compared to the covered codend sampling method (Eq (5)).

Uncertainty L50 (%)	Uniform fish distribution			No big fish			No medium fish			No small fish		
	Covered codend	Paired-gear	Ratio_n_	Covered codend	Paired-gear	Ratio_n_	Covered codend	Paired-gear	Ratio_n_	Covered codend	Paired-gear	Ratio_n_
1	5659	34255	6.05	4622	17540	3.79	10100	325505	32.23	6194	13544	2.19
2	1399	10175	7.27	1162	7310	6.29	2546	63716	25.03	1553	6061	3.90
3	618	5002	8.09	518	4381	8.46	1137	24543	21.59	691	3787	5.48
4	346	3022	8.73	292	3047	10.43	642	12472	19.43	389	2712	6.97
5	221	2045	9.25	187	2299	12.29	412	7378	17.91	249	2094	8.41
6	153	1486	9.71	130	1826	14.05	287	4804	16.74	173	1694	9.79
7	112	1134	10.13	96	1503	15.66	211	3343	15.84	127	1417	11.16
8	86	898	10.44	73	1270	17.40	162	2441	15.07	98	1214	12.39
9	67	730	10.90	58	1094	18.86	128	1850	14.45	77	1059	13.75
10	55	607	11.04	47	958	20.38	104	1444	13.88	63	937	14.87
11	45	514	11.42	39	849	21.77	86	1154	13.42	52	839	16.13
12	38	441	11.61	33	761	23.06	72	940	13.06	43	758	17.63
13	32	384	12.00	28	688	24.57	62	779	12.56	37	691	18.68
14	28	337	12.04	24	626	26.08	53	654	12.34	32	634	19.81
15	24	299	12.46	21	574	27.33	46	556	12.09	28	585	20.89
16	21	267	12.71	18	529	29.39	41	478	11.66	24	543	22.63
17	19	240	12.63	16	490	30.63	36	414	11.50	22	506	23.00
18	17	217	12.76	15	456	30.40	32	362	11.31	19	474	24.95
19	15	197	13.13	13	426	32.77	29	319	11.00	17	445	26.18
20	13	180	13.85	12	399	33.25	26	283	10.88	16	419	26.19

**Table 3:** Predicted number of fish needed to be measured to obtain an L50 value within a specific uncertainty (%) for the Mediterranean bottom trawl fishery with red mullet as reference species. Numbers are given for the covered codend and paired-gear sampling methods, and for each of the four fish size distribution scenarios investigated. Ratio_n_ quantifies the ratio of fish needed to be measured with the paired-gear sampling method compared to the covered codend sampling method (Eq (5)).

Uncertainty L50 (%)	Uniform fish distribution			No big fish			No medium fish			No small fish		
	Covered codend	Paired-gear	Ratio_n_	Covered codend	Paired-gear	Ratio_n_	Covered codend	Paired-gear	Ratio_n_	Covered codend	Paired-gear	Ratio_n_
1	2939	23095	7.86	3969	4469	1.13	2718	6899	2.54	2774	8041	2.90
2	737	6489	8.80	874	2422	2.77	1265	3531	2.79	692	3764	5.44
3	328	3088	9.41	360	1693	4.70	809	2387	2.95	307	2414	7.86
4	185	1823	9.85	192	1313	6.84	589	1807	3.07	172	1762	10.24
5	118	1212	10.27	118	1078	9.14	460	1457	3.17	110	1380	12.55
6	82	868	10.59	79	918	11.62	376	1222	3.25	77	1130	14.68
7	60	654	10.90	57	801	14.05	317	1053	3.32	56	954	17.04
8	46	512	11.13	42	712	16.95	274	925	3.38	43	824	19.16
9	37	413	11.16	33	642	19.45	240	826	3.44	34	725	21.32
10	30	340	11.33	26	585	22.50	214	746	3.49	27	646	23.93
11	25	286	11.44	21	537	25.57	193	680	3.52	23	582	25.30
12	21	244	11.62	17	498	29.29	175	625	3.57	19	529	27.84
13	18	211	11.72	15	464	30.93	160	579	3.62	16	484	30.25
14	15	184	12.27	12	434	36.17	148	539	3.64	14	447	31.93
15	13	162	12.46	11	409	37.18	137	504	3.68	12	414	34.50
16	12	144	12.00	9	386	42.89	127	474	3.73	11	386	35.09
17	10	129	12.90	8	366	45.75	119	447	3.76	9	361	40.11
18	9	116	12.89	7	348	49.71	112	423	3.78	8	339	42.38
19	8	105	13.13	6	332	55.33	105	401	3.82	8	320	40.00
20	7	96	13.71	6	317	52.83	100	382	3.82	7	302	43.14

Table 3 shows the same information for red mullet. With the exception of the fish distribution lacking medium sized fish, the ratio_n_ varied between 9.14 and 23.93 for ± 5% and ± 10% uncertainty in L50, which is similar to the result for the cod fishery. The ratio_n_ values for the case with no medium sized fish were 3.17 and 3.49 for ± 5% and ± 10% uncertainty in L50, respectively, and they were much lower than for all other cases. The actual number of fish that need be measured for this case was similar to the other three cases for the paired-gear method, but the number for this case was much larger than for the other three cases for the covered codend method (Table 3). The lower ratio_n_ values for this case are due to the greater uncertainty for the covered codend data for the red mullet population lacking medium sized fish compared to the other red mullet scenarios (see Fig 5). One potential explanation for the high covered codend uncertainty for this size distribution is that the data are based on a very small SR (1.25 cm), which suggests that very few length classes were present in the selective range. In a scenario in which medium sized fish are absent, this would create difficulties obtaining small uncertainty in L50. In any case, the data indicate that we would have to measure 10 times as many fish with the paired-gear method as with the covered codend method to obtain the same uncertainty level in L50 for the Mediterranean fishery. The actual numbers of fish that would need to be length measured to obtain an L50 with ± 5% uncertainty for the covered codend and paired-gear methods are 118 vs.1212, 118 vs. 1078, 460 vs. 1457, and 110 vs. 1380. These numbers are about half of those required for the cod fishery; the one exception is the scenario lacking medium sized fish, for which the values were at the same level. Similarly, for ± 10% uncertainty we would need to measure fewer fish to obtain the same uncertainty in L50 for the Mediterranean fishery compared to the cod fishery.

Depending on fishery and population size structure, these results suggest that around 120 to 250 fish would need to be length measured to obtain an uncertainty in L50 within ± 5% with the covered codend sampling method. Around 1080 to 2300 fish would be required for the paired-gear method, except for the case with no medium sized fish for which the required number often would be much higher. For an uncertainty level of ± 10%, the values would range from 30 to 65 fish for the covered codend method and from 340 to 960 for the paired-gear method. As a rule of thumb we could use that the paired-gear method requires catching and measuring from 8 to 15 times more fish to obtain the same precision in L50.

**Fig 8:**
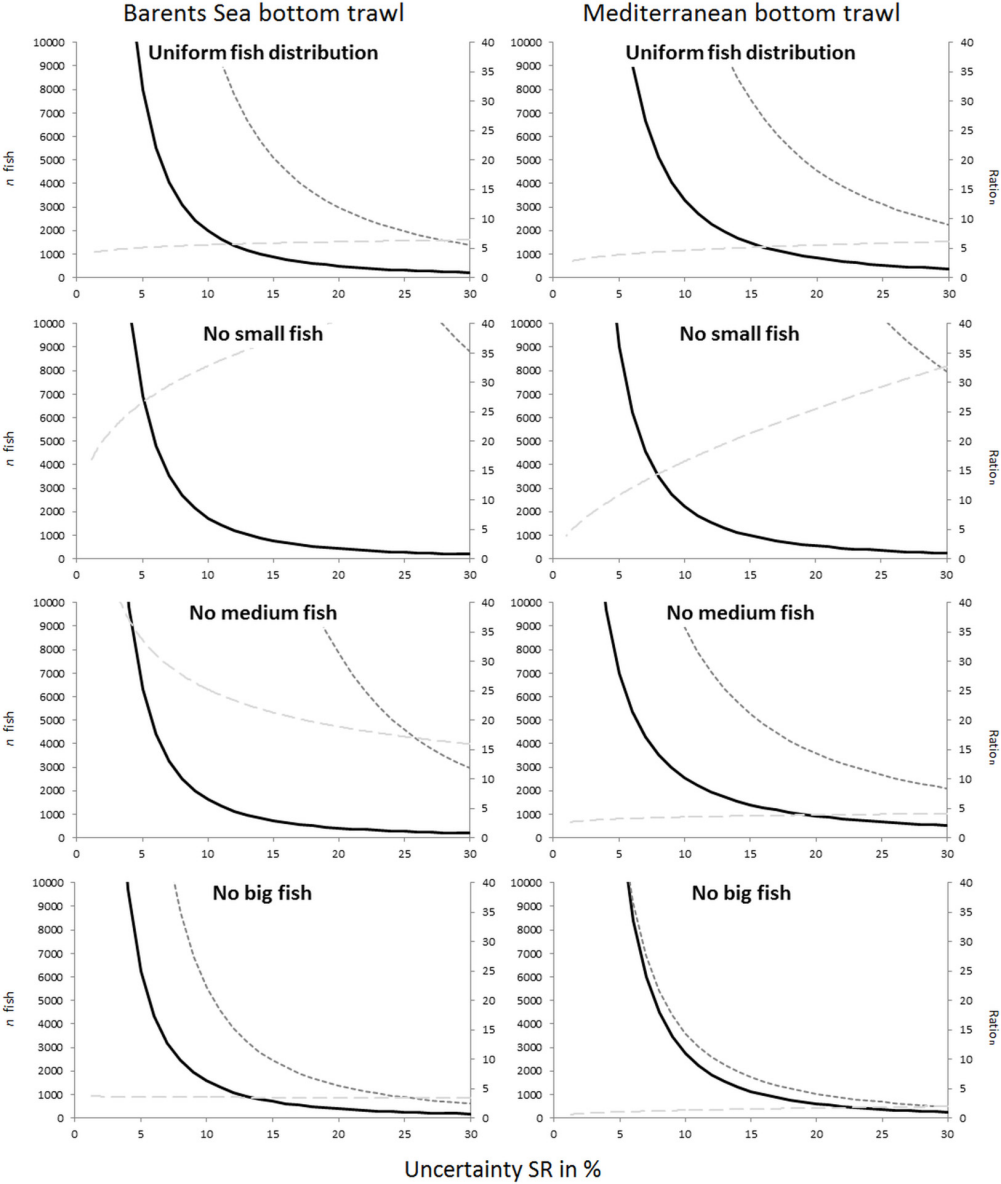
Predictions of number of fish that need to be measured to obtain an SR value (left axis) within specific levels of uncertainty (%) for the covered codend sampling method (solid black curve) and the paired-gear sampling method (stippled black curve). The stippled grey curve quantifies the ratio in number of fish, ratio_n_ (right axis), that need to be measured with the paired-gear method compared to the covered codend method to obtain the same level of uncertainty in L50. Predictions are shown for the four different fish size distribution scenarios investigated (from top): uniform, no small, no medium, and no big fish. The left column shows the results for the Barents Sea bottom trawl fishery, and the right column shows the results for the Mediterranean bottom trawl fishery represented by red mullet.

Fig 8 and Tables 4 and 5 show the number of fish that would need to be measured to obtain a specific expected uncertainty level in SR for the different cases investigated. As for L50, more fish would need to be caught and measured for the paired-gear method than for the covered codend method to obtain a specific uncertainty level. However, the ratio_n_ for SR varied more from case to case than for L50, and for the case with uniform fish size distribution the value was ~5.0 for both fisheries. In general the number of fish that would need to be measured to obtain uncertainty levels of ± 5% and ± 10% for SR was much higher than that for L50 for similar cases. Based on this result and the fact that SR generally is much smaller than L50, we considered levels of uncertainty for SR that correspond in cm to the ± 5% and ± 10% uncertainty levels for L50. This step was taken to provide guidelines for SR that are as realistic as possible.

**Table 4:** Predicted number of fish needed to be measured to obtain an SR value within a specific uncertainty (%) for the Barents Sea fishery targeting cod. Numbers are given for the covered codend and paired-gear sampling methods, and for each of the four fish size distribution scenarios investigated. Ratio_n_ quantifies the ratio of fish needed to be measured with the paired-gear sampling method compared to the covered codend sampling method (Eq (5)).

Uncertainty SR (%)	Uniform fish distribution			No big fish			No medium fish			No small fish		
	Covered codend	Paired-gear	Ratio_n_	Covered codend	Paired-gear	Ratio_n_	Covered codend	Paired-gear	Ratio_n_	Covered codend	Paired-gear	Ratio_n_
1	205248	847983	4.13	153146	571259	3.73	150150	9921432	66.08	172615	2806442	16.26
2	50742	229301	4.52	38604	141625	3.67	38366	1897255	49.45	43154	866834	20.09
3	22405	106698	4.76	17241	62637	3.63	17271	720875	41.74	19179	435989	22.73
4	12544	62005	4.94	9731	35111	3.61	9803	362808	37.01	10788	267741	24.82
5	8000	40698	5.09	6245	22411	3.59	6318	213004	33.71	6905	183426	26.56
6	5539	28852	5.21	4346	15529	3.57	4413	137851	31.24	4795	134665	28.08
7	4059	21571	5.31	3199	11388	3.56	3258	95419	29.29	3523	103702	29.44
8	3101	16767	5.41	2453	8705	3.55	2505	69379	27.70	2697	82698	30.66
9	2446	13425	5.49	1941	6868	3.54	1987	52378	26.36	2131	67732	31.78
10	1978	11005	5.56	1574	5556	3.53	1614	40732	25.24	1726	56655	32.82
11	1632	9194	5.63	1302	4586	3.52	1338	32445	24.25	1427	48204	33.78
12	1369	7802	5.70	1096	3850	3.51	1128	26361	23.37	1199	41594	34.69
13	1165	6708	5.76	934	3277	3.51	963	21777	22.61	1021	36318	35.57
14	1004	5833	5.81	806	2823	3.50	832	18247	21.93	881	32031	36.36
15	873	5121	5.87	703	2457	3.50	727	15477	21.29	767	28496	37.15
16	767	4534	5.91	618	2158	3.49	640	13267	20.73	674	25543	37.90
17	678	4044	5.96	548	1910	3.49	568	11480	20.21	597	23049	38.61
18	605	3630	6.00	489	1703	3.48	508	10016	19.72	533	20921	39.25
19	542	3278	6.05	439	1527	3.48	456	8804	19.31	478	19089	39.94
20	489	2976	6.09	397	1377	3.47	413	7789	18.86	432	17499	40.51
21	443	2714	6.13	360	1249	3.47	375	6933	18.49	391	16110	41.20
22	403	2486	6.17	328	1137	3.47	342	6204	18.14	357	14889	41.71
23	369	2286	6.20	301	1040	3.46	313	5580	17.83	326	13808	42.36
24	339	2110	6.22	276	954	3.46	288	5041	17.50	300	12847	42.82
25	312	1953	6.26	255	879	3.45	266	4573	17.19	276	11989	43.44
26	288	1814	6.30	236	812	3.44	246	4164	16.93	255	11218	43.99
27	267	1689	6.33	219	753	3.44	228	3806	16.69	237	10522	44.40
28	248	1577	6.36	203	700	3.45	213	3489	16.38	220	9893	44.97
29	231	1476	6.39	190	652	3.43	199	3209	16.13	205	9322	45.47
30	216	1385	6.41	177	609	3.44	186	2960	15.91	192	8802	45.84
45	95	644	6.78	79	269	3.41	84	1125	13.39	85	4427	52.08
90	24	174	7.25	20	67	3.35	21	215	10.24	21	1367	65.10

**Table 5:** Predicted number of fish needed to be measured to obtain an SR value within a specific uncertainty (%) for the Mediterranean bottom trawl fishery with red mullet as reference species. Numbers are given for the covered codend and paired-gear sampling methods, and for each of the four fish size distribution scenarios investigated. Ratio_n_ quantifies the ratio of fish needed to be measured with the paired-gear sampling method compared to the covered codend sampling method (Eq (5)).

Uncertainty SR (%)	Uniform fish distribution			No big fish			No medium fish			No small fish		
	Covered codend	Paired-gear	Ratio_n_	Covered codend	Paired-gear	Ratio_n_	Covered codend	Paired-gear	Ratio_n_	Covered codend	Paired-gear	Ratio_n_
1	319522	821833	2.57	423558	241649	0.57	73187	189120	2.58	231878	923178	3.98
2	80544	247211	3.07	92940	68212	0.73	26645	75605	2.84	57325	350633	6.12
3	35971	122429	3.40	38272	32548	0.85	14754	44221	3.00	25312	199028	7.86
4	20303	74362	3.66	20394	19255	0.94	9700	30225	3.12	14172	133174	9.40
5	13029	50512	3.88	12515	12814	1.02	7007	22500	3.21	9037	97515	10.79
6	9067	36827	4.06	8398	9188	1.09	5372	17678	3.29	6258	75593	12.08
7	6674	28193	4.22	5994	6935	1.16	4291	14417	3.36	4586	60951	13.29
8	5118	22368	4.37	4475	5435	1.21	3532	12083	3.42	3504	50581	14.44
9	4049	18238	4.50	3458	4384	1.27	2974	10340	3.48	2763	42909	15.53
10	3284	15194	4.63	2746	3617	1.32	2551	8995	3.53	2234	37037	16.58
11	2717	12881	4.74	2229	3040	1.36	2220	7929	3.57	1844	32421	17.58
12	2286	11078	4.85	1843	2593	1.41	1956	7067	3.61	1547	28711	18.56
13	1949	9643	4.95	1547	2241	1.45	1740	6357	3.65	1316	25674	19.51
14	1682	8481	5.04	1315	1958	1.49	1562	5764	3.69	1134	23150	20.41
15	1467	7525	5.13	1131	1726	1.53	1413	5261	3.72	987	21023	21.30
16	1290	6729	5.22	982	1534	1.56	1286	4830	3.76	866	19211	22.18
17	1144	6057	5.29	860	1374	1.60	1177	4458	3.79	767	17651	23.01
18	1021	5486	5.37	759	1237	1.63	1083	4134	3.82	683	16297	23.86
19	917	4995	5.45	674	1121	1.66	1001	3848	3.84	613	15112	24.65
20	828	4571	5.52	603	1021	1.69	929	3596	3.87	552	14067	25.48
21	751	4200	5.59	542	934	1.72	865	3371	3.90	501	13140	26.23
22	685	3875	5.66	489	858	1.75	808	3170	3.92	456	12314	27.00
23	627	3587	5.72	444	791	1.78	758	2989	3.94	417	11573	27.75
24	576	3332	5.78	404	732	1.81	712	2825	3.97	382	10905	28.55
25	531	3105	5.85	370	680	1.84	671	2677	3.99	352	10300	29.26
26	491	2901	5.91	339	633	1.87	634	2541	4.01	325	9751	30.00
27	456	2717	5.96	312	590	1.89	600	2418	4.03	302	9251	30.63
28	424	2551	6.02	289	553	1.91	569	2304	4.05	280	8793	31.40
29	396	2400	6.06	267	518	1.94	540	2200	4.07	261	8372	32.08
30	370	2264	6.12	248	487	1.96	514	2103	4.09	244	7985	32.73
45	165	1121	6.79	102	232	2.27	285	1230	4.32	108	4532	41.96
90	42	337	8.02	22	66	3.00	104	492	4.73	27	1721	63.74

Because the cod fishery data were based on simulation with L50 and SR set at 52 cm and 11.5 cm, respectively, uncertainty levels at ± 5% and ± 10% correspond respectively to ± 2.6 cm and ± 5.2 cm for L50 and about ± 23% and ± 45% for SR. In this study we used those levels of uncertainty as guidelines for the cod fishery regarding SR. Table 4 shows the expected numbers of fish that would need to be caught and measured for the four population structures in the cod fishery. These numbers were always higher for the paired-gear method than for the covered codend method. To obtain an expected uncertainty level in SR of ± 23%, we would have to measure 369 vs. 2286, 326 vs. 13808, 313 vs. 5500, and 301 vs. 1040 fish for the covered codend and paired-gear methods, respectively. For this level of uncertainty, the ratio_n_ was 6.20, 42.36, 17.57, and 3.46. For the ± 45% uncertainty level for SR, the number of fish that would need to be caught and measured would be 95 vs. 644, 85 vs. 4427, 84 vs. 1125, and 79 vs. 269, and the ratio_n_ values were 6.78, 52.08, 13.24, and 3.41.

For red mullet, the simulated data were based on an L50 value of 11.25 cm and a SR value of 1.25 cm. Thus, uncertainty levels at ± 5% and ± 10% for L50 would correspond to ± 0.5625 cm and ±1.125 cm, respectively, which would correspond to about ± 45% and ± 90% uncertainty for SR at 1.25 cm. We used those levels of uncertainty to develop guidelines for the red mullet fishery regarding SR. Table 5 shows the expected numbers of fish that would need to be caught and measured for the four population structures in the red mullet fishery. These numbers were again always higher for the paired-gear method than for the covered codend method. To obtain an expected uncertainty in SR of ± 45%, we would have to measure 165 vs. 1121, 108 vs. 4532, 285 vs. 1230, and 102 vs. 232 fish for the covered codend and paired-gear methods, respectively. For this level of uncertainty, the ratio_n_ values were 6.79, 41.96, 4.32, and 2.27, respectively. For the ± 90% uncertainty level for SR, the number of fish that would need to be caught and measured would be 42 vs. 337, 27 vs. 1721, 104 vs. 492, and 22 vs. 66, with ratio_n_ values of 8.02, 63.74, 4.73, and 3.0, respectively.

These results illustrate that it can be challenging to obtain SR values with low uncertainty, particularly when using the paired-gear sampling method. Considering the results obtained for both L50 and SR, it seems advisable to measure at least 300 fish with the covered codend method for a fishery such as the cod fishery and about 150 individuals for each species studied in a fishery such as the one represented by red mullet. The number of fish necessary is clearly much higher for the paired-gear method, and it will often be difficult to obtain SR values with a fair uncertainty level when using this type of gear. Thus, if the objective is to obtain SR with a decent uncertainty level, it is advisable to measure as many fish as possible when using the paired-gear method.

## Discussion

The dataset simulated in this study consisted of 279,000 hauls. By analyzing each of those hauls, we evaluated at the haul level the consequences of sampling effort and the necessity of measuring a certain number of fish to obtain L50 and SR estimates with specific limits of uncertainty. Additionally, the results presented here are also valuable at the multiple haul level. In a gear selectivity analysis that includes multiple hauls, which is the most likely scenario when the selective properties of a gear are to be assessed, there are two sources of uncertainty in the parameters estimated: between-haul variation and within-haul variation. As this study was conducted at the haul level, the between-haul variability was not considered. However, because this approach sets the between-haul variability to zero, the uncertainty levels shown in this study represent the lowest uncertainty threshold to be expected in a multiple haul selectivity analysis. Thus, the results described in this study are also useful as guidelines to determine the minimum number of fish that need to be caught and measured in trawl selectivity studies across multiple hauls.

Millar [21] investigated the effects of subsampling of catch data on size selectivity studies. He focused on the covered codend method and considered the efficiency of different sampling strategies and concluded that sampling an equal number of fish from the codend and cover provided the best overall efficiency among the strategies investigated. In another simulation-based study, Herrmann et al. [11] compared the covered codend and paired-gear estimation methods for estimation of selection parameters. However, that study did not investigate the effects of each method on uncertainty of confidence limits for L50 and SR. It also did not consider the effects of different fisheries and fish population structures. Thus, no previous study directly investigated the effects of data collection method (covered codend or paired-gear method) and size structure in the fished population on the uncertainty in selection parameters in terms of CIs. The fish population structure entering the fishing gear during a trawl haul is expected to vary from fishery to fishery and even from haul to haul. It is therefore impossible to define one specific fish population scenario to use in general to simulate the uncertainty in size selection that can be expected to be obtained during experimental fishing. Recognizing this challenge, we choose to base our investigation on a set of population size structures that might not individually be realistic for many fishing experiment cases. However, by examining this span of scenario's we hope to get an idea of the span in results that can be expected during different fishing situations.

The results presented herein clearly illustrate that increasing the effort of catching and measuring fish improves the results of selectivity analyses. Moreover, the covered codend method proved to be a more efficient sampling method in that fewer fish need to be caught and measured to obtain a specific level of uncertainty in the selection parameters L50 and SR. With equal sampling efforts, the uncertainty in L50 and SR would be completely different for the two sampling methods. In many cases, around 10 times more fish would need to caught with the paired-gear method compared to the covered codend method (for example 2000 vs. 200 fish) to obtain a specific uncertainty level. In scenarios where the possibilities to measure fish are limited the paired-gear method would also imply a risk for losing a substantial number of hauls due the impossibility of estimating the covariance matrix for those hauls.

Our results showed that when quantifying uncertainty in percentage of the parameter value it required far more fish to be length measured to obtain the same uncertainty level for SR as for L50. Considering that SR is a difference between length of fish with respectively 75% and 25% of being retained and therefore typically will have a value that is much smaller than the L50 value this makes sense. However, requiring the same percentage uncertainty level for SR as for L50 will result in a much smaller absolute uncertainty in SR than for L50. Based on such considerations and the fact that requiring the same percentage uncertainty level for SR as for L50 would lead to a unrealistic high number of fish needed to be length measured we proposed for SR to use as guidelines the number of fished needed to be measured to obtain the same level of absolute uncertainty as for L50.

Considering the difference in performance between the covered codend and paired-gear methods, it seems illogical to choose the paired-gear method for any fishing gear selectivity study. However, there are many situations in which it is impractical or even impossible to use the covered codend method. Thus, it is important to develop guidelines for sampling effort for both methods. The results provided herein are intended to act as guidelines for practitioners planning size selectivity trials. The variation in results obtained between the two fisheries investigated and the different fish population structure scenarios considered in this study illustrate that it is not possible to provide an exact number of fish that need to be caught and measured to obtain selection parameter values within planned limits of uncertainty. However, the values provided in this study provide guidelines that give a rough idea of what to expect when using either of the two sampling methods routinely used by fisheries scientists in selectivity studies.

## Supporting Information

S1 Appendix (DOCX)
