# Supplementary material for: How Many Fish Need to Be Measured to Effectively Evaluate Trawl Selectivity?
Source: PLoS One. 2016 Aug 25;11(8):e0161512. doi: 10.1371/journal.pone.0161512 (PMC4999221; doi:10.1371/journal.pone.0161512)
Supplement: S1 Appendix — (DOCX) [file pone.0161512.s001.docx]

**S1 Appendix.** The tables (F–M) in this appendix show the uncertainty predictions for L50 and SR in % versus the number of fish measured for the two fisheries (Barents Sea bottom trawl cod fishery and Mediterranean bottom trawl fishery represented by red mullet) and four fish size distribution scenarios (uniform size distribution, no small, no medium, and no big fish) included in this study. *n* fish shows the number of fish length measured in each case. Bias L50 and bias SR were calculated as described in Materials and Methods. The values in brackets show the 2.5 and 97.5 percentile limits of the uncertainty in % for each of the cases (see section 2.4). *n* hauls show the number of hauls for which it was actually possible to estimate out of the simulated 1000 hauls. Predictions are given for both the covered codend sampling method (above) and the paired-gear sampling method (below).

**Table F.** Barents Sea bottom trawl fishery results for uniform cod size distribution.

|  | *n* fish | Bias L50 (%) | Uncertainty L50 (%) | Bias SR in % | Uncertainty SR (%) | *n* hauls |
| --- | --- | --- | --- | --- | --- | --- |
| Covered codend | 100 | 0.30 | 7.36 (5.29 ̶ 9.23) | -5.05 | 43.73 (31.52 ̶ 54.95) | 1000 |
|  | 200 | 0.12 | 5.25 (4.33 ̶ 6.1) | -2.91 | 31.11 (25.43 ̶ 36.38) | 1000 |
|  | 300 | 0.09 | 4.29 (3.67 ̶ 4.87) | -1.95 | 25.46 (21.81 ̶ 28.86) | 1000 |
|  | 400 | -0.03 | 3.74 (3.31 ̶ 4.13) | -1.02 | 22.15 (19.62 ̶ 24.67) | 1000 |
|  | 500 | 0.11 | 3.34 (3 ̶ 3.67) | -1.39 | 19.77 (17.62 ̶ 21.81) | 1000 |
|  | 600 | 0.06 | 3.05 (2.75 ̶ 3.31) | -1.52 | 18.03 (16.29 ̶ 19.71) | 1000 |
|  | 700 | 0.01 | 2.83 (2.6 ̶ 3.04) | -1.18 | 16.72 (15.33 ̶ 18) | 1000 |
|  | 800 | 0.05 | 2.65 (2.46 ̶ 2.85) | -0.96 | 15.66 (14.48 ̶ 16.86) | 1000 |
|  | 900 | 0.00 | 2.49 (2.33 ̶ 2.65) | -0.90 | 14.76 (13.81 ̶ 15.71) | 1000 |
|  | 1000 | 0.04 | 2.36 (2.21 ̶ 2.5) | -0.99 | 13.99 (13.14 ̶ 14.86) | 1000 |
|  | 1100 | 0.01 | 2.26 (2.12 ̶ 2.38) | -0.63 | 13.37 (12.57 ̶ 14.19) | 1000 |
|  | 1200 | 0.02 | 2.16 (2.04 ̶ 2.27) | -0.72 | 12.8 (12.1 ̶ 13.52) | 1000 |
|  | 1300 | 0.01 | 2.07 (1.96 ̶ 2.17) | -0.81 | 12.28 (11.62 ̶ 12.95) | 1000 |
|  | 1400 | 0.02 | 2 (1.9 ̶ 2.1) | -0.62 | 11.86 (11.24 ̶ 12.48) | 1000 |
|  | 1500 | -0.01 | 1.93 (1.85 ̶ 2.02) | -0.48 | 11.46 (10.95 ̶ 12) | 1000 |
|  | 1600 | 0.01 | 1.87 (1.81 ̶ 1.94) | -0.81 | 11.08 (10.67 ̶ 11.52) | 1000 |
|  | 1700 | 0.03 | 1.82 (1.75 ̶ 1.88) | -0.60 | 10.76 (10.38 ̶ 11.14) | 1000 |
|  | 1800 | 0.00 | 1.76 (1.71 ̶ 1.83) | -0.64 | 10.45 (10.1 ̶ 10.86) | 1000 |
|  | 1900 | 0.05 | 1.72 (1.65 ̶ 1.77) | -0.70 | 10.17 (9.81 ̶ 10.48) | 1000 |
|  | 2000 | 0.05 | 1.68 (1.63 ̶ 1.73) | -0.65 | 9.92 (9.62 ̶ 10.19) | 1000 |
|  | 2100 | 0.02 | 1.63 (1.6 ̶ 1.67) | -0.52 | 9.68 (9.43 ̶ 10) | 1000 |
|  | 2200 | 0.01 | 1.6 (1.56 ̶ 1.63) | -0.56 | 9.46 (9.24 ̶ 9.71) | 1000 |
|  | 2300 | 0.03 | 1.56 (1.52 ̶ 1.6) | -0.59 | 9.25 (9.05 ̶ 9.43) | 1000 |
| Paired-gear | 100 | 1.07 | 30.18 (7.26 ̶ 92.09) | -15.59 | 114.39 (29.10 ̶ 329.38) | 725 |
|  | 200 | -0.03 | 18.89 (7.94 ̶ 39.70) | -9.43 | 85.76 (31.46 ̶ 164.26) | 932 |
|  | 300 | -1.28 | 14.83 (7.21 ̶ 28.78) | -11.37 | 74.18 (33.85 ̶ 109.51) | 988 |
|  | 400 | -0.68 | 12.29 (7.00 ̶ 19.17) | -7.06 | 59.06 (34.10 ̶ 88.00) | 990 |
|  | 500 | -0.64 | 10.63 (6.98 ̶ 16.08) | -6.35 | 50.15 (34.00 ̶ 74.19) | 1000 |
|  | 600 | -0.65 | 9.81 (6.75 ̶ 14.33) | -4.82 | 46.47 (32.38 ̶ 66.95) | 1000 |
|  | 700 | -0.73 | 9.02 (6.58 ̶ 12.35) | -4.77 | 42.63 (31.43 ̶ 57.81) | 1000 |
|  | 800 | -0.56 | 8.42 (6.29 ̶ 10.98) | -3.79 | 39.78 (30.19 ̶ 52.48) | 1000 |
|  | 900 | -0.77 | 7.87 (6.1 ̶ 10.13) | -4.80 | 37.18 (29.05 ̶ 47.43) | 1000 |
|  | 1000 | -0.87 | 7.48 (5.87 ̶ 9.31) | -4.98 | 35.41 (28.00 ̶ 44.19) | 1000 |
|  | 1100 | -0.64 | 7.11 (5.85 ̶ 8.75) | -4.36 | 33.61 (27.62 ̶ 41.14) | 1000 |
|  | 1200 | -0.71 | 6.85 (5.73 ̶ 8.27) | -3.91 | 32.44 (27.05 ̶ 39.14) | 1000 |
|  | 1300 | -0.82 | 6.56 (5.52 ̶ 7.88) | -4.22 | 31.07 (26.10 ̶ 37.14) | 1000 |
|  | 1400 | -0.65 | 6.30 (5.37 ̶ 7.38) | -4.10 | 29.80 (25.33 ̶ 35.14) | 1000 |
|  | 1500 | -0.71 | 6.12 (5.33 ̶ 7.06) | -3.88 | 28.94 (25.14 ̶ 33.43) | 1000 |
|  | 1600 | -0.73 | 5.89 (5.19 ̶ 6.65) | -3.93 | 27.89 (24.67 ̶ 31.62) | 1000 |
|  | 1700 | -0.66 | 5.73 (5.12 ̶ 6.42) | -3.65 | 27.10 (24.10 ̶ 30.48) | 1000 |
|  | 1800 | -0.74 | 5.55 (5.04 ̶ 6.13) | -3.92 | 26.29 (23.81 ̶ 29.14) | 1000 |

**Table G.** Barents Sea bottom trawl fishery results for a distribution with no small cod.

|  | *n* fish | Bias L50 (%) | Uncertainty L50 (%) | Bias SR (%) | Uncertainty SR (%) | *n* hauls |
| --- | --- | --- | --- | --- | --- | --- |
| Covered codend | 100 | 0.73 | 7.89 (5.63 ̶ 10.44) | -0.75 | 41.51 (28.95 ̶ 56.86) | 1000 |
|  | 200 | 0.53 | 5.59 (4.56 ̶ 6.83) | 0.87 | 29.40 (22.86 ̶ 36.67) | 1000 |
|  | 300 | 0.37 | 4.54 (3.83 ̶ 5.33) | 1.08 | 23.92 (19.71 ̶ 28.29) | 1000 |
|  | 400 | 0.35 | 3.94 (3.44 ̶ 4.44) | 1.46 | 20.74 (17.62 ̶ 23.81) | 1000 |
|  | 500 | 0.34 | 3.53 (3.17 ̶ 3.88) | 1.63 | 18.57 (16.19 ̶ 20.95) | 1000 |
|  | 600 | 0.33 | 3.22 (2.92 ̶ 3.48) | 1.69 | 16.96 (15.14 ̶ 18.86) | 1000 |
|  | 700 | 0.34 | 2.98 (2.77 ̶ 3.21) | 1.65 | 15.69 (14.19 ̶ 17.33) | 1000 |
| Paired-gear | 100 | 2.65 | 68.91 (6.71 ̶ 333.22) | -17.35 | 337.86 (29.76 ̶ 1383.22) | 661 |
|  | 200 | 1.93 | 40.61 (4.65 ̶ 232.46) | 6.65 | 274.70 (24.03 ̶ 1326.44) | 791 |
|  | 300 | 1.47 | 31.33 (3.9 ̶ 200.94) | 11.93 | 233.03 (22.64 ̶ 1096.89) | 869 |
|  | 400 | 0.45 | 19.24 (3.84 ̶ 101.27) | 7.69 | 186.21 (21.66 ̶ 825.78) | 913 |
|  | 500 | 0.22 | 17.05 (3.65 ̶ 99.18) | 13.08 | 179.82 (24.52 ̶ 811.63) | 932 |
|  | 600 | 0.30 | 14.93 (3.92 ̶ 59.83) | 14.59 | 161.36 (27.75 ̶ 669.54) | 965 |
|  | 700 | 0.11 | 12.81 (3.5 ̶ 53.64) | 14.98 | 153.43 (25.79 ̶ 670.84) | 978 |
|  | 800 | 0.19 | 11.62 (4.15 ̶ 36.44) | 12.85 | 138.32 (32.93 ̶ 536.76) | 991 |
|  | 900 | -0.46 | 9.32 (4.10 ̶ 22.37) | 7.89 | 121.35 (33.52 ̶ 365.07) | 992 |
|  | 1000 | -0.32 | 8.76 (3.98 ̶ 22.5) | 10.81 | 114.30 (35.60 ̶ 325.18) | 998 |
|  | 1100 | -0.23 | 8.46 (4.21 ̶ 17.97) | 9.27 | 108.51 (38.47 ̶ 295.08) | 999 |
|  | 1200 | -0.27 | 7.39 (4.29 ̶ 14.56) | 7.07 | 97.41 (41.52 ̶ 240.29) | 1000 |
|  | 1300 | -0.04 | 7.31 (4.33 ̶ 12.77) | 8.77 | 95.21 (44.00 ̶ 243.52) | 1000 |
|  | 1400 | -0.39 | 6.70 (4.23 ̶ 10.92) | 5.91 | 87.82 (41.52 ̶ 172.95) | 1000 |
|  | 1500 | -0.19 | 6.33 (4.19 ̶ 10.04) | 6.35 | 83.34 (43.62 ̶ 166.10) | 1000 |
|  | 1600 | -0.32 | 6.01 (4.12 ̶ 9.35) | 4.45 | 79.24 (41.81 ̶ 146.67) | 1000 |
|  | 1700 | -0.33 | 5.86 (4.02 ̶ 8.83) | 5.15 | 77.58 (43.14 ̶ 138.95) | 1000 |
|  | 1800 | -0.32 | 5.68 (4.12 ̶ 8.29) | 5.38 | 74.88 (44.95 ̶ 134.00) | 1000 |
|  | 1900 | -0.30 | 5.47 (4.04 ̶ 7.6) | 4.47 | 72.13 (44.29 ̶ 117.71) | 1000 |
|  | 2000 | -0.31 | 5.29 (3.98 ̶ 7.04) | 3.77 | 69.54 (44.67 ̶ 108.48) | 1000 |
|  | 2100 | -0.29 | 5.19 (4.00 ̶ 6.94) | 4.81 | 68.31 (45.14 ̶ 106.38) | 1000 |
|  | 2200 | -0.32 | 5.00 (4.04 ̶ 6.33) | 3.36 | 65.57 (46.00 ̶ 95.14) | 1000 |
|  | 2300 | -0.35 | 4.86 (3.94 ̶ 5.92) | 2.80 | 63.70 (46.57 ̶ 88.19) | 1000 |
|  | 2400 | -0.28 | 4.76 (4.02 ̶ 5.67) | 3.19 | 62.15 (47.24 ̶ 81.90) | 1000 |
|  | 2500 | -0.31 | 4.66 (4.04 ̶ 5.46) | 3.35 | 61.08 (48.29 ̶ 77.81) | 1000 |
|  | 2600 | -0.31 | 4.54 (4.08 ̶ 5.08) | 2.75 | 59.38 (49.33 ̶ 71.52) | 1000 |

**Table H.** Barents Sea bottom trawl fishery results for a distribution with no medium-sized cod.

|  | *n* fish | Bias L50 (%) | Uncertainty L50 (%) | Bias SR (%) | Uncertainty SR (%) | *n* hauls |
| --- | --- | --- | --- | --- | --- | --- |
| Covered Codend | 100 | 0.46 | 10.29 (1.6 ̶ 8.14) | -7.24 | 45.42 (37.15 ̶ 54.94) | 962 |
|  | 200 | -0.05 | 7.16 (0.43 ̶ 6.46) | -3.64 | 31.19 (27.14 ̶ 35.24) | 1000 |
|  | 300 | 0.07 | 5.85 (0.25 ̶ 5.38) | -2.05 | 25.38 (22.67 ̶ 28.00) | 1000 |
|  | 400 | 0.08 | 5.09 (0.2 ̶ 4.73) | -0.82 | 22.00 (20.10 ̶ 23.90) | 1000 |
|  | 500 | -0.06 | 4.55 (0.14 ̶ 4.29) | -0.14 | 19.64 (18.19 ̶ 21.14) | 1000 |
|  | 600 | 0.09 | 4.14 (0.12 ̶ 3.90) | -0.82 | 17.87 (16.48 ̶ 19.14) | 1000 |
|  | 700 | -0.13 | 3.83 (0.10 ̶ 3.65) | 0.08 | 16.55 (15.43 ̶ 17.62) | 1000 |
|  | 800 | 0.05 | 3.59 (0.08 ̶ 3.44) | -0.05 | 15.49 (14.57 ̶ 16.38) | 1000 |
|  | 900 | -0.04 | 3.38 (0.07 ̶ 3.23) | 0.47 | 14.61 (13.81 ̶ 15.43) | 1000 |
|  | 1000 | 0.01 | 3.2 (0.06 ̶ 3.08) | 0.04 | 13.83 (13.24 ̶ 14.48) | 1000 |
|  | 1100 | -0.06 | 3.05 (0.06 ̶ 2.94) | -0.03 | 13.18 (12.57 ̶ 13.71) | 1000 |
|  | 1200 | -0.06 | 2.93 (0.05 ̶ 2.83) | 0.28 | 12.64 (12.1 ̶ 13.14) | 1000 |
|  | 1300 | -0.05 | 2.81 (0.04 ̶ 2.73) | 0.44 | 12.13 (11.71 ̶ 12.57) | 1000 |
|  | 1400 | -0.06 | 2.71 (0.04 ̶ 2.63) | 0.43 | 11.69 (11.24 ̶ 12.1) | 1000 |
|  | 1500 | -0.04 | 2.62 (0.04 ̶ 2.56) | 0.37 | 11.29 (10.95 ̶ 11.71) | 1000 |
|  | 1600 | -0.11 | 2.53 (0.03 ̶ 2.46) | 0.70 | 10.94 (10.57 ̶ 11.33) | 1000 |
|  | 1700 | -0.06 | 2.46 (0.03 ̶ 2.4) | 0.72 | 10.61 (10.29 ̶ 10.95) | 1000 |
|  | 1800 | -0.07 | 2.39 (0.03 ̶ 2.35) | 0.64 | 10.31 (10 ̶ 10.57) | 1000 |
|  | 1900 | -0.07 | 2.32 (0.02 ̶ 2.27) | 0.63 | 10.04 (9.81 ̶ 10.29) | 1000 |
|  | 2000 | -0.08 | 2.26 (0.02 ̶ 2.23) | 0.59 | 9.78 (9.52 ̶ 10) | 1000 |
|  | 2100 | -0.10 | 2.21 (0.02 ̶ 2.17) | 0.65 | 9.54 (9.33 ̶ 9.71) | 1000 |
|  | 2200 | -0.08 | 2.16 (0.02 ̶ 2.13) | 0.64 | 9.32 (9.14 ̶ 9.52) | 1000 |
| Paired-gear | 100 | 2.34 | 33.65 (25.84 ̶ 8.1) | -16.21 | 123.08 (30.95 ̶ 319.95) | 674 |
|  | 200 | -0.88 | 23.31 (15.56 ̶ 6.4) | -12.90 | 100.39 (28.57 ̶ 173.37) | 892 |
|  | 300 | -1.59 | 18.57 (8.25 ̶ 7.08) | -11.38 | 76.91 (31.71 ̶ 137.92) | 974 |
|  | 400 | -1.63 | 16.5 (7.99 ̶ 6.9) | -10.01 | 68.21 (31.85 ̶ 113.9) | 992 |
|  | 500 | -1.67 | 15.08 (5.74 ̶ 6.96) | -9.84 | 62.41 (34.08 ̶ 105.74) | 998 |
|  | 600 | -1.51 | 14.24 (3.94 ̶ 7.44) | -7.76 | 57.55 (35.71 ̶ 90.48) | 1000 |
|  | 700 | -1.87 | 13.31 (3.58 ̶ 7.31) | -8.67 | 53.94 (34.1 ̶ 86.86) | 1000 |
|  | 800 | -1.90 | 12.58 (3.17 ̶ 7.37) | -8.43 | 51.05 (34.1 ̶ 78.57) | 1000 |
|  | 900 | -2.07 | 12.01 (2.73 ̶ 7.46) | -8.20 | 48.75 (33.81 ̶ 71.81) | 1000 |
|  | 1000 | -1.86 | 11.81 (3.37 ̶ 7.5) | -7.68 | 47.81 (33.43 ̶ 73.05) | 1000 |
|  | 1100 | -1.90 | 11.32 (2.37 ̶ 7.5) | -7.60 | 45.74 (33.24 ̶ 67.62) | 1000 |
|  | 1200 | -2.23 | 10.83 (2.07 ̶ 7.52) | -8.51 | 43.97 (33.14 ̶ 60.48) | 1000 |
|  | 1300 | -2.14 | 10.55 (1.9 ̶ 7.5) | -8.05 | 42.73 (32.48 ̶ 57.62) | 1000 |
|  | 1400 | -1.89 | 10.23 (1.58 ̶ 7.56) | -6.98 | 41.35 (32.38 ̶ 54.67) | 1000 |
|  | 1500 | -2.04 | 9.99 (1.45 ̶ 7.42) | -7.28 | 40.45 (31.62 ̶ 52.67) | 1000 |
|  | 1600 | -1.93 | 9.74 (1.28 ̶ 7.65) | -6.81 | 39.34 (32.48 ̶ 50.48) | 1000 |
|  | 1700 | -1.98 | 9.55 (1.16 ̶ 7.38) | -6.89 | 38.55 (31.05 ̶ 48) | 1000 |
|  | 1800 | -1.96 | 9.24 (0.97 ̶ 7.5) | -6.92 | 37.28 (31.52 ̶ 44.67) | 1000 |

**Table I.** Barents Sea bottom trawl fishery results for a distribution with no big cod.

|  | *n* fish | Bias L50 (%) | Uncertainty L50 (%) | Bias SR (%) | Uncertainty SR (%) | *n* hauls |
| --- | --- | --- | --- | --- | --- | --- |
| Covered codend | 100 | -0.35 | 6.87 (4.83 ̶ 9.69) | 0.28 | 40.20 (26.48 ̶ 59.52) | 1000 |
|  | 200 | -0.42 | 4.83 (3.81 ̶ 6.08) | 0.85 | 28.10 (21.14 ̶ 36.57) | 1000 |
|  | 300 | -0.33 | 3.94 (3.27 ̶ 4.71) | 1.26 | 22.99 (18.48 ̶ 28.38) | 1000 |
|  | 400 | -0.38 | 3.40 (2.92 ̶ 3.94) | 0.97 | 19.80 (16.38 ̶ 23.52) | 1000 |
|  | 500 | -0.30 | 3.05 (2.69 ̶ 3.46) | 1.46 | 17.78 (15.43 ̶ 20.67) | 1000 |
|  | 600 | -0.29 | 2.79 (2.5 ̶ 3.12) | 1.62 | 16.25 (14.19 ̶ 18.67) | 1000 |
|  | 700 | -0.32 | 2.58 (2.35 ̶ 2.83) | 1.49 | 15.01 (13.33 ̶ 16.86) | 1000 |
|  | 800 | -0.28 | 2.41 (2.21 ̶ 2.63) | 1.68 | 14.07 (12.67 ̶ 15.62) | 1000 |
|  | 900 | -0.33 | 2.27 (2.12 ̶ 2.42) | 1.44 | 13.22 (12.1 ̶ 14.48) | 1000 |
|  | 1000 | -0.28 | 2.16 (2.04 ̶ 2.31) | 1.69 | 12.58 (11.52 ̶ 13.71) | 1000 |
|  | 1100 | -0.28 | 2.06 (1.94 ̶ 2.17) | 1.61 | 11.99 (11.05 ̶ 12.95) | 1000 |
| Paired-gear | 100 | -4.72 | 55.18 (7.66 ̶ 336.73) | -23.29 | 73.99 (28 ̶ 141.23) | 844 |
|  | 200 | -3.77 | 37.67 (7.8 ̶ 278.77) | -14.97 | 51.76 (30.58 ̶ 84.15) | 964 |
|  | 300 | -3.87 | 27.91 (8.37 ̶ 170.51) | -12.74 | 42.68 (28.99 ̶ 62.61) | 992 |
|  | 400 | -4.53 | 19.82 (8.42 ̶ 58.77) | -12.09 | 37.27 (27.62 ̶ 51.16) | 997 |
|  | 500 | -5.29 | 15.10 (8.10 ̶ 34.31) | -12.45 | 33.05 (25.71 ̶ 44.10) | 1000 |

**Table J.** Mediterranean Sea bottom trawl fishery results for uniform red mullet size distribution.

|  | *n* fish | Bias L50 (%) | Uncertainty L50 (%) | Bias SR (%) | Uncertainty SR (%) | *n* hauls |
| --- | --- | --- | --- | --- | --- | --- |
| Covered codend | 100 | 0.12 | 5.59 (3.11 ̶ 7.38) | -10.30 | 60.11 (36.8 ̶ 151.2) | 998 |
|  | 200 | 0.07 | 3.78 (2.76 ̶ 4.62) | -3.63 | 40.13 (30.4 ̶ 48) | 1000 |
|  | 300 | 0.10 | 3.1 (2.49 ̶ 3.64) | -2.80 | 32.96 (27.2 ̶ 37.6) | 1000 |
|  | 400 | -0.02 | 2.69 (2.22 ̶ 3.11) | -1.94 | 28.63 (24.8 ̶ 32) | 1000 |
|  | 500 | 0.09 | 2.41 (2.04 ̶ 2.67) | -1.61 | 25.62 (22.4 ̶ 28.8) | 1000 |
|  | 600 | 0.05 | 2.21 (1.96 ̶ 2.49) | -1.10 | 23.45 (20.8 ̶ 25.6) | 1000 |
|  | 700 | 0.07 | 2.05 (1.78 ̶ 2.22) | -1.05 | 21.7 (20 ̶ 24) | 1000 |
|  | 800 | 0.00 | 1.91 (1.69 ̶ 2.13) | -0.95 | 20.31 (18.4 ̶ 21.6) | 1000 |
|  | 900 | 0.04 | 1.8 (1.6 ̶ 1.96) | -0.79 | 19.18 (17.6 ̶ 20.8) | 1000 |
|  | 1000 | 0.04 | 1.72 (1.6 ̶ 1.87) | -0.46 | 18.22 (16.8 ̶ 19.2) | 1000 |
|  | 1100 | 0.04 | 1.63 (1.51 ̶ 1.78) | -0.61 | 17.34 (16 ̶ 18.4) | 1000 |
|  | 1200 | 0.05 | 1.57 (1.42 ̶ 1.69) | -0.11 | 16.65 (15.2 ̶ 17.6) | 1000 |
|  | 1300 | 0.05 | 1.5 (1.42 ̶ 1.6) | -0.49 | 15.98 (15.2 ̶ 16.8) | 1000 |
|  | 1400 | -0.01 | 1.45 (1.33 ̶ 1.51) | -0.32 | 15.39 (14.4 ̶ 16) | 1000 |
|  | 1500 | 0.03 | 1.4 (1.33 ̶ 1.51) | -0.54 | 14.87 (14.4 ̶ 15.2) | 1000 |
|  | 1600 | 0.01 | 1.36 (1.24 ̶ 1.42) | -0.01 | 14.42 (13.6 ̶ 15.2) | 1000 |
|  | 1700 | 0.02 | 1.32 (1.24 ̶ 1.42) | 0.13 | 14.02 (13.6 ̶ 14.4) | 1000 |
|  | 1800 | 0.02 | 1.28 (1.24 ̶ 1.33) | -0.38 | 13.59 (12.8 ̶ 14.4) | 1000 |
|  | 1900 | 0.02 | 1.24 (1.16 ̶ 1.33) | -0.24 | 13.22 (12.8 ̶ 13.6) | 1000 |
|  | 2000 | 0.03 | 1.22 (1.16 ̶ 1.24) | -0.24 | 12.86 (12.8 ̶ 13.6) | 1000 |
|  | 2100 | 0.03 | 1.18 (1.16 ̶ 1.24) | -0.33 | 12.64 (12 ̶ 12.8) | 1000 |
|  | 2200 | 0.03 | 1.16 (1.16 ̶ 1.16) | -0.17 | 12.22 (12 ̶ 12.8) | 1000 |
|  | 2300 | 0.02 | 1.15 (1.07 ̶ 1.16) | -0.11 | 12.01 (12 ̶ 12) | 1000 |
|  | 2400 | 0.02 | 1.1 (1.07 ̶ 1.16) | -0.16 | 11.89 (11.2 ̶ 12) | 1000 |
|  | 2500 | 0.03 | 1.07 (1.07 ̶ 1.16) | -0.13 | 11.44 (11.2 ̶ 12) | 1000 |
| Paired-gear | 100 | -2.19 | 22.48 (3.45 ̶ 143.31) | -31.12 | 181.7 (32.66 ̶ 561.64) | 993 |
|  | 200 | -3.11 | 12 (2.84 ̶ 27.29) | -21.34 | 118.79 (30.4 ̶ 317.6) | 1000 |
|  | 300 | -2.38 | 10.24 (3.02 ̶ 19.02) | -10.06 | 100.08 (39.2 ̶ 260) | 1000 |
|  | 400 | -2.25 | 8.89 (3.64 ̶ 16.62) | -5.61 | 83.79 (40.8 ̶ 213.6) | 1000 |
|  | 500 | -2.22 | 7.85 (3.82 ̶ 12.44) | -3.02 | 71.63 (42.4 ̶ 162.4) | 1000 |
|  | 600 | -1.80 | 7.23 (4.62 ̶ 10.58) | 1.26 | 63.3 (41.6 ̶ 97.6) | 1000 |
|  | 700 | -1.80 | 6.76 (4.8 ̶ 9.69) | 2.78 | 59.06 (41.6 ̶ 84.8) | 1000 |
|  | 800 | -1.90 | 6.23 (4.62 ̶ 8.53) | 2.29 | 53.26 (40 ̶ 73.6) | 1000 |
|  | 900 | -1.70 | 5.9 (4.53 ̶ 7.73) | 4.01 | 50.51 (38.4 ̶ 66.4) | 1000 |
|  | 1000 | -1.67 | 5.62 (4.44 ̶ 7.2) | 4.44 | 47.93 (37.6 ̶ 61.6) | 1000 |
|  | 1100 | -1.58 | 5.33 (4.27 ̶ 6.76) | 5.61 | 45.41 (36.8 ̶ 58.4) | 1000 |
|  | 1200 | -1.57 | 5.09 (4.27 ̶ 6.22) | 5.59 | 43.14 (36 ̶ 52.8) | 1000 |
|  | 1300 | -1.54 | 4.87 (4.18 ̶ 5.78) | 6.26 | 41.23 (35.2 ̶ 48.8) | 1000 |
|  | 1400 | -1.56 | 4.69 (4 ̶ 5.42) | 6.38 | 39.74 (34.4 ̶ 46.4) | 1000 |
|  | 1500 | -1.54 | 4.52 (4 ̶ 5.24) | 6.54 | 38.31 (33.6 ̶ 44) | 1000 |
|  | 1600 | -1.55 | 4.36 (3.91 ̶ 4.98) | 6.60 | 36.98 (32.8 ̶ 41.6) | 1000 |
|  | 1700 | -1.55 | 4.24 (3.82 ̶ 4.71) | 6.70 | 35.94 (32 ̶ 40) | 1000 |

**Table K.** Mediterranean Sea bottom trawl fishery results for a distribution with no small red mullet.

|  | *n* fish | Bias L50 (%) | Uncertainty L50 (%) | Bias SR (%) | Uncertainty SR (%) | *n* hauls |
| --- | --- | --- | --- | --- | --- | --- |
| Covered codend | 100 | -0.20 | 5.24 (3.64 ̶ 7.29) | -1.94 | 46.83 (30.4 ̶ 68) | 1000 |
|  | 200 | -0.40 | 3.7 (2.93 ̶ 4.62) | -0.42 | 33.14 (25.6 ̶ 42.4) | 1000 |
|  | 300 | -0.37 | 3.03 (2.58 ̶ 3.56) | 0.00 | 27.1 (22.4 ̶ 32.8) | 1000 |
|  | 400 | -0.46 | 2.62 (2.31 ̶ 2.93) | 0.43 | 23.58 (20 ̶ 27.2) | 1000 |
|  | 500 | -0.46 | 2.34 (2.13 ̶ 2.58) | 0.47 | 21.08 (18.4 ̶ 24) | 1000 |
| Paired-gear | 100 | 3.70 | 53.89 (2.84 ̶ 457.49) | 34.30 | 568.26 (32.8 ̶ 4021.8) | 750 |
|  | 200 | 0.58 | 31.15 (3.44 ̶ 215.36) | 45.94 | 449.78 (38.68 ̶ 2795.72) | 854 |
|  | 300 | -0.74 | 21.8 (2.22 ̶ 98.36) | 31.12 | 347.3 (41.6 ̶ 1856.88) | 916 |
|  | 400 | -0.70 | 20.17 (3.58 ̶ 62.84) | 34.44 | 290.3 (44 ̶ 1514.5) | 945 |
|  | 500 | -1.33 | 14.17 (3.56 ̶ 50.6) | 33.89 | 261.67 (44 ̶ 1090.92) | 974 |
|  | 600 | -1.40 | 10.21 (3.38 ̶ 32.3) | 26.73 | 196.87 (40.54 ̶ 784) | 987 |
|  | 700 | -1.47 | 8.03 (3.56 ̶ 22.57) | 19.82 | 169.24 (45.6 ̶ 510.24) | 991 |
|  | 800 | -1.20 | 6.74 (3.2 ̶ 16.18) | 16.87 | 143.39 (42.36 ̶ 412.2) | 998 |
|  | 900 | -1.29 | 6.67 (3.29 ̶ 16.01) | 19.51 | 144.41 (43.2 ̶ 447.24) | 998 |
|  | 1000 | -1.25 | 5.88 (3.11 ̶ 13.37) | 15.70 | 125.35 (42.3 ̶ 357.5) | 995 |
|  | 1100 | -1.32 | 5.41 (3.11 ̶ 10.4) | 12.87 | 116.87 (44 ̶ 280.8) | 1000 |
|  | 1200 | -1.37 | 5.22 (3.02 ̶ 10.76) | 14.14 | 115.66 (43.2 ̶ 299.2) | 1000 |
|  | 1300 | -1.35 | 4.85 (2.93 ̶ 9.33) | 10.15 | 104.27 (43.2 ̶ 240.8) | 1000 |
|  | 1400 | -1.36 | 4.68 (3.02 ̶ 8.44) | 11.69 | 103.19 (45.6 ̶ 232.8) | 1000 |
|  | 1500 | -1.28 | 4.38 (2.93 ̶ 7.2) | 8.81 | 94.78 (44.8 ̶ 194.4) | 1000 |
|  | 1600 | -1.33 | 4.27 (2.84 ̶ 6.93) | 10.90 | 94.5 (44.8 ̶ 188) | 1000 |
|  | 1700 | -1.36 | 4.05 (2.84 ̶ 6.22) | 8.20 | 89.03 (44.8 ̶ 170.4) | 1000 |
|  | 1800 | -1.35 | 3.94 (2.84 ̶ 5.78) | 9.02 | 86.54 (47.2 ̶ 156.8) | 1000 |
|  | 1900 | -1.38 | 3.84 (2.84 ̶ 5.6) | 9.21 | 84.92 (48 ̶ 150.4) | 1000 |
|  | 2000 | -1.30 | 3.65 (2.76 ̶ 5.24) | 7.04 | 79.73 (47.2 ̶ 140) | 1000 |
|  | 2100 | -1.34 | 3.56 (2.76 ̶ 4.71) | 7.11 | 77.84 (48.8 ̶ 122.4) | 1000 |
|  | 2200 | -1.37 | 3.45 (2.76 ̶ 4.44) | 5.97 | 75.5 (48.8 ̶ 118.4) | 1000 |
|  | 2300 | -1.36 | 3.39 (2.76 ̶ 4.27) | 6.86 | 74.46 (49.6 ̶ 111.2) | 1000 |
|  | 2400 | -1.33 | 3.3 (2.76 ̶ 4) | 6.47 | 72.28 (50.4 ̶ 104) | 1000 |
|  | 2500 | -1.36 | 3.22 (2.76 ̶ 3.73) | 5.89 | 70.75 (52.8 ̶ 93.6) | 1000 |
|  | 2600 | -1.37 | 3.14 (2.84 ̶ 3.56) | 5.46 | 69.07 (54.4 ̶ 88) | 1000 |

**Table L.** Mediterranean Sea bottom trawl fishery results for a distribution with medium-sized red mullet.

|  | *n* fish | Bias L50 (%) | Uncertainty L50 (%) | Bias SR (%) | Uncertainty SR (%) | *n* hauls |
| --- | --- | --- | --- | --- | --- | --- |
| Covered codend | 100 | 0.04 | 69.11 (3.82 ̶ 1424.36) | -28.22 | 164.94 (35.2 ̶ 1822.4) | 976 |
|  | 200 | -0.54 | 10.35 (3.64 ̶ 7.64) | -17.46 | 53.86 (32.8 ̶ 181.6) | 1000 |
|  | 300 | -0.68 | 4.07 (3.38 ̶ 4.62) | -12.00 | 33.92 (28.8 ̶ 38.4) | 1000 |
|  | 400 | -0.84 | 3.49 (3.11 ̶ 3.91) | -10.37 | 28.68 (24.8 ̶ 32) | 1000 |
|  | 500 | -0.80 | 3.12 (2.84 ̶ 3.47) | -10.68 | 25.42 (22.4 ̶ 28) | 1000 |
|  | 600 | -0.82 | 2.85 (2.58 ̶ 3.11) | -9.52 | 23.22 (20.8 ̶ 25.6) | 1000 |
|  | 700 | -0.85 | 2.64 (2.4 ̶ 2.84) | -8.81 | 21.57 (20 ̶ 23.2) | 1000 |
|  | 800 | -0.89 | 2.46 (2.31 ̶ 2.67) | -8.48 | 20.1 (18.4 ̶ 21.6) | 1000 |
|  | 900 | -0.91 | 2.32 (2.13 ̶ 2.49) | -8.36 | 18.92 (17.6 ̶ 20) | 1000 |
|  | 1000 | -0.90 | 2.21 (2.04 ̶ 2.31) | -8.45 | 17.94 (16.8 ̶ 19.2) | 1000 |
|  | 1100 | -0.91 | 2.1 (1.96 ̶ 2.22) | -7.78 | 17.17 (16 ̶ 18.4) | 1000 |
|  | 1200 | -0.95 | 2.02 (1.96 ̶ 2.13) | -7.89 | 16.4 (15.2 ̶ 17.6) | 1000 |
|  | 1300 | -0.91 | 1.93 (1.87 ̶ 2.04) | -8.25 | 15.74 (15.2 ̶ 16.8) | 1000 |
|  | 1400 | -0.97 | 1.87 (1.78 ̶ 1.96) | -7.11 | 15.21 (14.4 ̶ 16) | 1000 |
|  | 1500 | -0.92 | 1.8 (1.69 ̶ 1.87) | -7.83 | 14.65 (13.6 ̶ 15.2) | 1000 |
|  | 1600 | -0.90 | 1.74 (1.69 ̶ 1.78) | -7.79 | 14.21 (13.6 ̶ 14.4) | 1000 |
|  | 1700 | -0.92 | 1.69 (1.6 ̶ 1.78) | -7.63 | 13.74 (13.6 ̶ 14.4) | 1000 |
|  | 1800 | -0.90 | 1.64 (1.6 ̶ 1.69) | -7.68 | 13.42 (12.8 ̶ 13.6) | 1000 |
|  | 1900 | -0.94 | 1.6 (1.51 ̶ 1.69) | -7.52 | 12.96 (12.8 ̶ 13.6) | 1000 |
|  | 2000 | -0.91 | 1.56 (1.51 ̶ 1.6) | -7.56 | 12.74 (12 ̶ 12.8) | 1000 |
|  | 2100 | -0.93 | 1.51 (1.51 ̶ 1.6) | -7.60 | 12.38 (12 ̶ 12.8) | 1000 |
|  | 2200 | -0.91 | 1.5 (1.42 ̶ 1.51) | -7.50 | 12.02 (12 ̶ 12.8) | 1000 |
|  | 2300 | -0.92 | 1.44 (1.42 ̶ 1.51) | -7.42 | 11.98 (11.2 ̶ 12) | 1000 |
|  | 2400 | -0.93 | 1.42 (1.42 ̶ 1.42) | -7.34 | 11.54 (11.2 ̶ 12) | 1000 |
| Paired-gear | 100 | 5.26 | 77.3 (3.29 ̶ 1550.67) | -29.13 | 258.97 (30.78 ̶ 2002.32) | 899 |
|  | 200 | 2.98 | 50.94 (3.47 ̶ 162.62) | -23.44 | 191.04 (29.6 ̶ 1170.4) | 980 |
|  | 300 | 2.78 | 25.1 (3.29 ̶ 114.93) | -14.08 | 155.46 (28 ̶ 514.72) | 996 |
|  | 400 | 2.65 | 21.33 (3.64 ̶ 38.31) | -8.72 | 113.8 (26.4 ̶ 369.6) | 1000 |
|  | 500 | 3.13 | 15.87 (3.56 ̶ 30.22) | -4.71 | 92.54 (25.6 ̶ 245.6) | 1000 |
|  | 600 | 3.38 | 9.89 (4.53 ̶ 22.58) | 0.86 | 74.08 (31.2 ̶ 206.4) | 1000 |
|  | 700 | 3.97 | 8.46 (5.07 ̶ 19.29) | 6.06 | 64.19 (36.8 ̶ 171.2) | 1000 |
|  | 800 | 4.47 | 7.67 (5.16 ̶ 15.73) | 9.32 | 57.82 (39.2 ̶ 114.4) | 1000 |
|  | 900 | 4.34 | 7.76 (5.16 ̶ 14.67) | 9.81 | 58.18 (39.2 ̶ 114.4) | 1000 |
|  | 1000 | 4.53 | 7.04 (4.89 ̶ 14.04) | 11.87 | 52.21 (36.8 ̶ 97.6) | 1000 |
|  | 1100 | 4.66 | 6.55 (4.71 ̶ 9.69) | 12.56 | 48.35 (35.2 ̶ 68.8) | 1000 |
|  | 1200 | 5.13 | 6.25 (4.8 ̶ 8.36) | 16.22 | 45.69 (36 ̶ 57.6) | 1000 |
|  | 1300 | 5.15 | 5.86 (4.62 ̶ 7.2) | 16.42 | 43.05 (35.2 ̶ 50.4) | 1000 |
|  | 1400 | 5.43 | 5.46 (4.71 ̶ 6.58) | 18.16 | 40.13 (35.2 ̶ 46.4) | 1000 |
|  | 1500 | 5.28 | 5.31 (4.62 ̶ 6.49) | 17.31 | 38.86 (34.4 ̶ 45.6) | 1000 |
|  | 1600 | 5.37 | 5.11 (4.44 ̶ 5.96) | 18.25 | 37.44 (33.6 ̶ 42.4) | 1000 |
|  | 1700 | 5.45 | 4.91 (4.44 ̶ 5.51) | 18.83 | 36.06 (32.8 ̶ 40) | 1000 |

**Table M.** Mediterranean Sea bottom trawl fishery results for a distribution with no big red mullet (simulated with 5 times the original size distribution).

|  | *n* fish | Bias L50 (%) | Uncertainty L50 (%) | Bias SR (%) | Uncertainty SR (%) | *n* hauls |
| --- | --- | --- | --- | --- | --- | --- |
| Covered codend | 100 | 0.38 | 5.76 (3.91 ̶ 8.36) | 3.62 | 48.42 (32.00 ̶ 71.20) | 1000 |
|  | 200 | 0.26 | 4.00 (3.11 ̶ 5.07) | 2.70 | 33.66 (24.80 ̶ 43.20) | 1000 |
|  | 300 | 0.37 | 3.29 (2.67 ̶ 4.09) | 3.71 | 27.78 (22.4 ̶ 34.40) | 1000 |
|  | 400 | 0.36 | 2.85 (2.4 ̶ 3.47) | 3.58 | 24.02 (20 ̶ 29.60) | 1000 |
|  | 500 | 0.34 | 2.55 (2.13 ̶ 3.11) | 3.40 | 21.5 (18.4 ̶ 26.40) | 1000 |
|  | 600 | 0.38 | 2.34 (2.04 ̶ 3.11) | 3.98 | 19.78 (16.8 ̶ 25.60) | 1000 |
|  | 700 | 0.34 | 2.15 (1.87 ̶ 2.58) | 3.33 | 18.22 (16 ̶ 21.6) | 1000 |
|  | 800 | 0.41 | 2.03 (1.78 ̶ 2.58) | 4.17 | 17.17 (15.2 ̶ 20.8) | 1000 |
|  | 900 | 0.37 | 1.91 (1.69 ̶ 2.40) | 3.86 | 16.14 (14.4 ̶ 20) | 1000 |
|  | 1000 | 0.38 | 1.82 (1.60 ̶ 2.40) | 3.91 | 15.38 (13.6 ̶ 20) | 1000 |
|  | 1100 | 0.36 | 1.73 (1.51 ̶ 2.31) | 3.68 | 14.62 (12.8 ̶ 19.2) | 1000 |
|  | 1200 | 0.34 | 1.67 (1.51 ̶ 2.31) | 3.39 | 14.1 (12.8 ̶ 20) | 1000 |
|  | 1300 | 0.35 | 1.60 (1.42 ̶ 2.22) | 3.53 | 13.54 (12 ̶ 19.2) | 1000 |
|  | 1400 | 0.37 | 1.56 (1.42 ̶ 2.22) | 3.83 | 13.17 (12 ̶ 18.4) | 1000 |
|  | 1500 | 0.40 | 1.51 (1.33 ̶ 2.13) | 3.89 | 12.8 (11.2 ̶ 17.6) | 1000 |
|  | 1600 | 0.39 | 1.49 (1.33 ̶ 2.13) | 3.88 | 12.62 (11.2 ̶ 18.4) | 1000 |
|  | 1700 | 0.38 | 1.46 (1.24 ̶ 2.04) | 3.82 | 12.3 (10.4 ̶ 17.6) | 1000 |
|  | 1800 | 0.35 | 1.43 (1.24 ̶ 2.04) | 3.71 | 12.03 (10.4 ̶ 16.8) | 1000 |
|  | 1900 | 0.40 | 1.41 (1.24 ̶ 1.96) | 4.02 | 11.9 (10.4 ̶ 16.8) | 1000 |
|  | 2000 | 0.39 | 1.38 (1.16 ̶ 1.96) | 3.86 | 11.65 (9.6 ̶ 16.8) | 1000 |
|  | 2100 | 0.40 | 1.38 (1.16 ̶ 1.96) | 3.98 | 11.66 (9.6 ̶ 16) | 1000 |
|  | 2200 | 0.42 | 1.38 (1.16 ̶ 1.87) | 4.12 | 11.66 (9.6 ̶ 16) | 1000 |
|  | 2300 | 0.38 | 1.37 (1.16 ̶ 1.87) | 3.86 | 11.56 (9.6 ̶ 16) | 1000 |
|  | 2400 | 0.39 | 1.39 (1.07 ̶ 1.87) | 4.00 | 11.69 (9.6 ̶ 15.2) | 1000 |
| Paired-gear | 100 | -1.54 | 69.20 (4.00 ̶ 431.06) | -40.63 | 76.54 (29.6 ̶ 264.04) | 999 |
|  | 200 | -2.08 | 35.41 (4.00 ̶ 242.33) | -33.70 | 46.88 (25.6 ̶ 85.66) | 999 |
|  | 300 | -2.30 | 23.77 (4.00 ̶ 185.24) | -31.37 | 37.26 (24 ̶ 52.8) | 1000 |
|  | 400 | -3.03 | 15.41 (3.91 ̶ 128.89) | -31.50 | 32.42 (22.4 ̶ 44.8) | 1000 |
|  | 500 | -2.93 | 12.19 (3.82 ̶ 68.18) | -29.87 | 29.34 (21.6 ̶ 39.2) | 1000 |
|  | 600 | -3.41 | 9.21 (3.56 ̶ 24.71) | -30.46 | 26.66 (20 ̶ 33.6) | 1000 |
|  | 700 | -3.59 | 7.70 (3.47 ̶ 16.98) | -30.88 | 24.4 (19.2 ̶ 31.2) | 1000 |
|  | 800 | -3.37 | 6.91 (3.64 ̶ 15.47) | -29.56 | 23.09 (18.4 ̶ 28) | 1000 |
|  | 900 | -3.69 | 5.84 (3.29 ̶ 11.20) | -30.22 | 21.72 (17.6 ̶ 26.4) | 1000 |
|  | 1000 | -3.62 | 5.52 (3.29 ̶ 10.49) | -30.10 | 20.54 (16.8 ̶ 24.8) | 1000 |
|  | 1100 | -3.65 | 5.25 (3.29 ̶ 9.78) | -29.82 | 19.72 (16.8 ̶ 23.2) | 1000 |
